# Novel Tyrosinase and α-Glucosidase Inhibitors: 1,3-Bisbenzylphenylphenol and Congeners as Cosmetic Whitening Agents Based on Natural Products

**DOI:** 10.3390/molecules31030573

**Published:** 2026-02-06

**Authors:** Meng-Fei Wanyan, Xing Wu, Hui-Xiang Yang, Liang Tu, Qian Chen, Zhi-Hui Dong, Yu-Ting Tian, Xiao Lv, Qiong Chen, Hui-Hui Shen, Ting-Ting Deng, Zheng-Hui Li, Xian Wang, Rong Huang, Yong-Sheng Zheng, Ji-Kai Liu

**Affiliations:** 1 School of Pharmaceutical Sciences, South-Central Minzu University, Wuhan 430074, China; wymf666666@163.com (M.-F.W.); ajoc199515@163.com (L.T.); chenqian@cque.edu.cn (Q.C.); sophia163900@163.com (Z.-H.D.); bc25019007@mail.ustc.edu.cn (Y.-T.T.); alvlvx@163.com (X.L.); joanchen787@163.com (Q.C.); qwepoi202512@163.com (T.-T.D.); lizhenghui@mail.scuec.edu.cn (Z.-H.L.); 2 Anhui Province Key Laboratory of Bioactive Natural Products, School of Pharmacy, Anhui University of Chinese Medicine, Hefei 230012, China; wuxing812@outlook.com (X.W.); yanghuixiang@ahtcm.edu.cn (H.-X.Y.); 3 Shenzhen Moore Vaporization Health & Medical Technology Co., Ltd., Wuhan 430074, China; huihuishen2025@163.com

**Keywords:** skin-whitening agents, tyrosinase inhibition, α-Glucosidase inhibition, 1,3-bisbenzylphenylphenolic compounds, aquatic plant *Ottelia acuminata* var. *acuminata*

## Abstract

New diarylheptene polyphenols with α-glucosidase inhibitory activity were previously isolated and reported from the aquatic plant *Ottelia acuminata* var. *acuminata*. It was used as the template in the present research, and a series of 1,3-bisbenzylphenylphenolic compounds were designed and synthesized. The tyrosinase, α-glucosidase inhibitory effects, antioxidant properties, and whitening effects of these compounds were investigated. Of them, the representative compounds **1** and **2** inhibited the two target enzymes (tyrosinase and α-glucosidase) engaged in skin whitening and aging with comparable IC_50_ values to the reference drugs as well as antioxidant activities. They showed potent whitening efficacy in zebrafish. In particular, compound **1** had whitening-effect rates of 31% at a concentration of 0.0001% (m/m), and 52% at a concentration of 0.0002% (m/m). Both compounds had more superior whitening efficacy than the commercially available whitening agent phenylethylresorcinol (377), which was used as a positive control. Compounds **1** and **2** did not show any genotoxicity and skin phototoxicity at the test concentrations, and they show promise as new skin-whitening agents.

## 1. Introduction

The skin is the largest organ in our body and the first barrier to the invasion of microorganisms. It protects our body from external invasions while maintaining thermal adjustment and transmitting the sense of touch [1]. Increased skin pigmentation occurs secondary to various factors, including age, endocrine disorders, hormone levels, inflammation, and environmental exposures, including dermatologic conditions caused by ultraviolet (UV) and infrared radiation. The pigmentation is generated by increased production and deposition of melanin in the epidermis [2].

Melanocytes are located in the basal layer of the skin that separates the dermis and the epidermis. Approximately 36 keratinocytes surround one melanocyte [3]. In response to ultraviolet B (UVB) radiation, melanocytes synthesize melanin through a course called melanogenesis. The melanin synthesized in the melanosomes is carried to neighboring keratinocytes in the epidermis [4]. There are two main types of melanin, red/yellow pheomelanin and brown/black eumelanin, which differ not only in color but also in shape, size, and particle packing. The biosynthesis of melanin can begin with either L-tyrosine or L-dihydroxyphenylalanine (L-DOPA), which is oxidized to dopaquinone and is the common pathway for the production of eumelanin and pheomelanin [5]. The first step in the melanogenesis process is catalyzed by the key tyrosinase enzyme, which oxidizes L-tyrosine to dopaquinone. The resulting quinone is used to synthesize eumelanin and pheomelanin. This step, the production of dopaquinone, is the rate-limiting step in melanin synthesis, as all other sequential reactions can proceed automatically at physiological pH. Due to the importance of tyrosinase in melanin synthesis, direct inhibition of tyrosinase catalytic activity becomes the most prominent and successful target of melanogenesis inhibitors. Most commercially available cosmetic or skin-whitening agents are tyrosinase inhibitors [4,6].

At present, most of the skin-whitening and brightening agents on the market are based on the inhibition of tyrosinase, such as hydroquinone, arbutin, kojic acid, tretinoin, 4-butylresorcinol, etc. Hydroquinone has a very simple phenolic structure. It is widely distributed in nature and has been universally used as an effective lightener in cosmetic formulations. Most countries have banned the use of hydroquinone in cosmetics due to its side effects, such as carcinogenesis, long-lasting depigmentation, and the increased incidence of ochronosis with long-term use [5]. Arbutin is a hydroquinone derivative, and is one of the most ordinary components in skin-whitening creams worldwide [7]. Its cytotoxic effect on melanocytes leads to strong skin irritation, and long-term use may cause permanent white spots on the skin, and it has been included in the list of banned ingredients in cosmetics by many countries [5]. Kojic acid is a natural organic acid that is a by-product of certain species of fungi, such as *Aspergillus* and *Penicillium*. It inhibits tyrosinase by capturing copper ions in the active site of tyrosinase, thereby preventing melanin synthesis [8]. The most common retinoid in skin-whitening creams is tretinoin. It acts in a variety of ways: inhibiting tyrosinase, interfering with the transfer of melanin to keratinocytes, and accelerating pigment loss by stimulating epidermal turnover [9]. 4-Butylresorcinol, a powerful tyrosinase inhibitor and tyrosinase-related protein-1 inhibitor, inhibits the production of new melanin in melanocytes [10]. Combining ingredients that target one or more steps in the melanogenesis pathway is an appealing approach for pigmentation control for the potential synergies it offers [11]. Therefore, the discovery of novel, safe and effective whitening agents from natural products is very important for whitening cosmetics.

Previously, Xu Gang’s group reported a series of diarylheptene polyphenolic compounds with significant α-glucosidase inhibitory activity and potential therapeutic value for diabetes mellitus from *Ottelia acuminata* var. *acuminata*, an aquatic plant of edible vegetables of the Bai ethnic group in Dali, Yunnan [12]. Studies have shown that tyrosinase is a protein with a sugar chain, and during its maturation process, the original sugar chain must undergo a series of modifications and cleavage before it can be converted into a mature tyrosinase and exert its normal biological efficacy, and α-glucosidase I and II are the key enzymes in the process of modification and cleavage of the sugar chain [13]. When α-glucosidase is inhibited, the modification of sugar chains on glycoproteins is blocked from producing active tyrosinase, and there is a corresponding reduction in melanin formation [14]. Therefore, reducing tyrosinase maturation by inhibiting α-glucosidase for the purpose of reducing melanin production and skin pigmentation is a novel possible route to skin whitening and for combating age spots.

**Figure 1:**
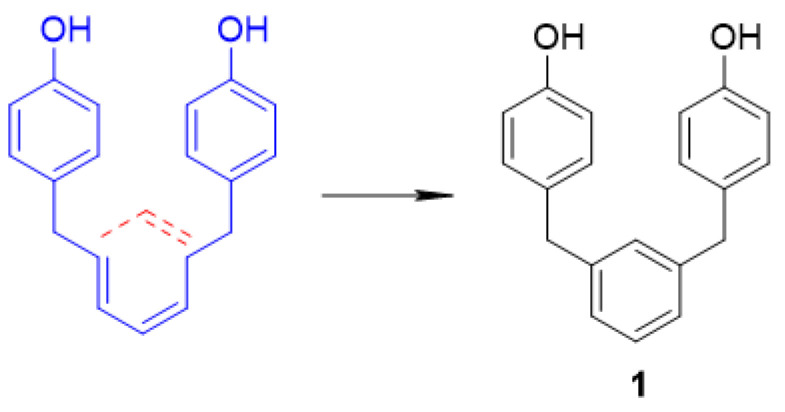
The natural product from *Ottelia acuminata* var. *acuminata* as a template.

We took these kind of natural products as lead compounds, and used them as a template to simplify and replace their open-chain flexible molecular structures into rigid benzene ring polyphenolic structural analogs by using conformational restriction and bioisosterism strategies (Figure 1), and then we synthesized a series of 1,3-bis-benzylbenzil phenolic compounds, and their tyrosinase, α-glucosidase inhibitory activities, antioxidant activities and cellular melanogenesis inhibitory activities were investigated. Compound **1** was synthesized previously as an intermediate of polymer materials [15]; there are no existing reports on its biological activities. This type of compound was first discovered for its tyrosinase, α-glucosidase inhibitory activities, and antioxidant activities. It showed the potent inhibitory activities for cellular melanin synthesis, and has good prospects for application in whitening, and anti-aging treatments.

## 2. Results

In the present investigation, 31 compounds with 1,3-bis-benzylbenzil core structure were designed and synthesized. The 1,3-bisbenzylphenylphenol derivatives were prepared in accordance with methods known from the literature [15]. Their tyrosinase, α-glucosidase inhibitory activities, and antioxidant activities were screened and evaluated.

**Figure 2:**
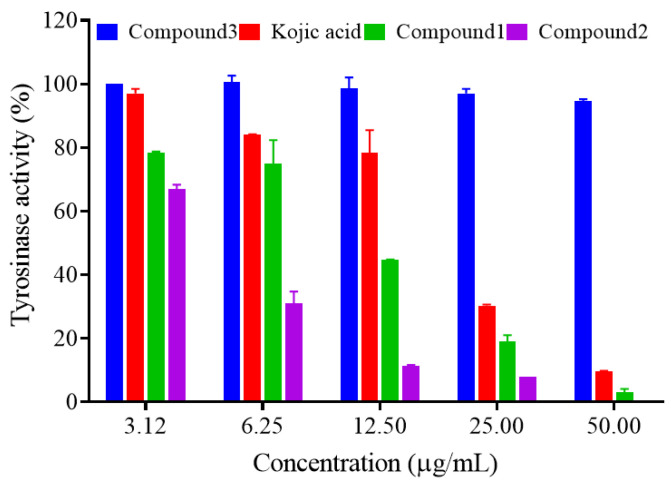
Effect of compounds **1**–**3** and the positive kojic acid on tyrosinase activity.

Compounds **1** and **2** and the positive control kojic acid [8,9] showed dose-dependent inhibition of tyrosinase activity, and compound **2** showed the best inhibitory effect. The IC_50_ of the half-inhibitory concentrations of compounds **1** and **2** and kojic acid on tyrosinase were 10.43 μg/mL (i.e., 35.96 μM), 4.36 μg/mL (i.e., 13.68 μM) and 18.61 μg/mL (i.e., 131.6 μM), respectively, whereas compound **3** had no activity at any of the tested concentrations (IC_50_ > 50 μg/mL). The results showed that compounds **1** and **2** had good tyrosinase inhibitory activity, with tyrosinase inhibitory activity effects 3.7 and 9.6 times more effective than that of kojic acid, respectively (Figure 2).

**Figure 3:**
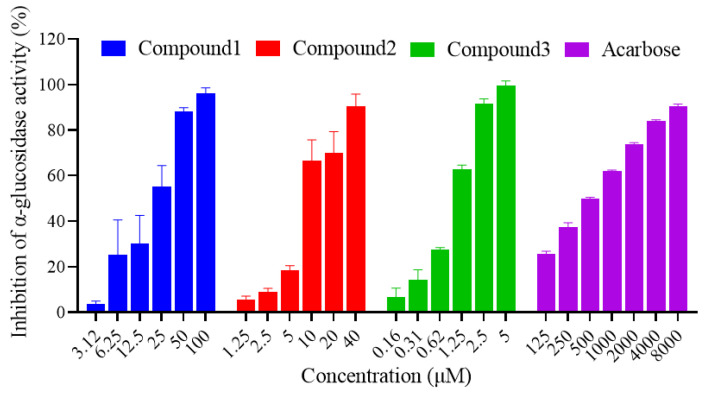
Inhibition of α-glucosidase activity by compounds **1**–**3** and positive control acarbose.

All three compounds and the positive control drug acarbose produced dose-dependent inhibition of α-glucosidase activity. The IC_50_ of compounds **1**–**3** and acarbose [16] on α-glucosidase were calculated to be 21.77, 8.91, 0.95 and 481.7 μM, respectively, after applying Graphpad Prism 8.0 software for non-linear fitting. The above experimental results showed that all three compounds had good inhibitory activity against α-glucosidase, and the inhibitory effect was better than that of the positive control acarbose (Figure 3).

**Figure 4:**
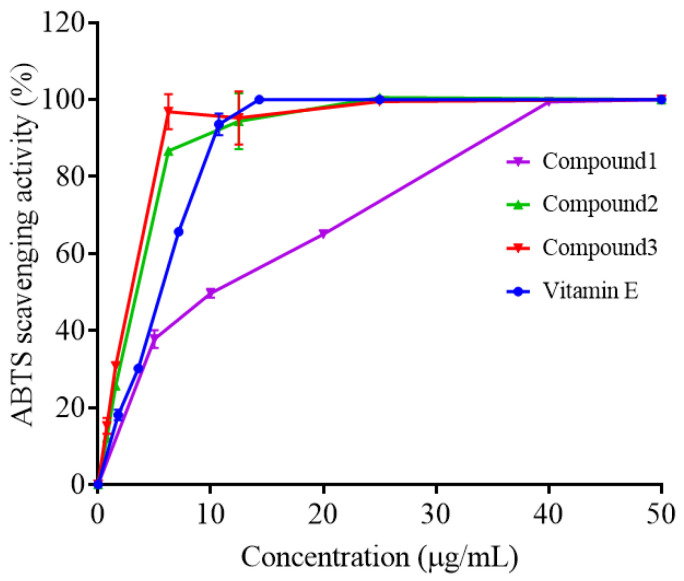
Scavenging effect of the compounds **1**–**3** and the positive control vitamin E on ABTS free radicals.

The compounds to be tested also showed good scavenging effect on ABTS radicals in the tested concentration range [17]. The IC_50_ (half maximal inhibitory concentration) of compounds **1**–**3** on the scavenging of ABTS radicals was calculated to be 6.78 μg/mL (i.e., 23.22 μM), 2.56 μg/mL (i.e., 8.02 μM), and 2.10 μg/mL (i.e., 6.49 μM), respectively, and the IC_50_ value of the positive control drug vitamin E was 4.75 μg/mL (i.e., 11.05 μM). The above results showed that compounds **1**–**3** have good free radical scavenging ability, among which compounds **2** and **3** have better antioxidant activity than the positive control vitamin E, which is expected to help cells resist oxidative stress damage and delay skin aging (Figure 4, Table 1).

**Figure 5:**
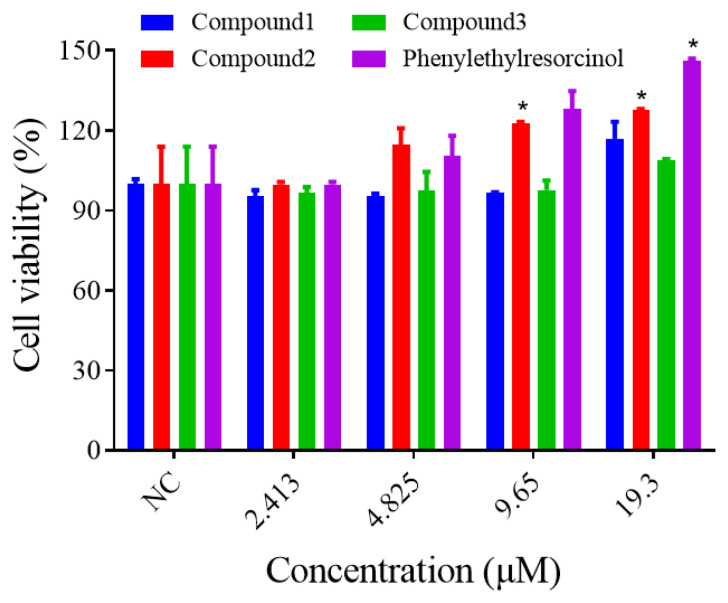
Effect of the compounds **1**–**3** and the positive control phenylethylresorcinol on B16 cell viability. * *p*< 0.05.

For cellular activity assay, the statistical analysis software was SPSS 31.0, and the comparison between the test samples and the negative control was done by independent samples *t*-test. All of the above statistical analyses were two-tailed tests, and the significance level was α = 0.05. * *p* < 0.05, * indicates that there is a significant difference between the test samples under the effect of this concentration compared with the negative control, and the experimental results are shown in Figure 5. As can be seen from Figure 5, the three compounds at a concentration of 19.3 μmol/L showed >90% cell viability and no cytotoxicity.

In the assay of cellular melanin synthesis inhibition, the statistical analysis software was SPSS, and independent samples *t*-test was used for comparison between test samples and negative control. All of the above statistical analyses were performed as two-tailed tests with a significance level of α = 0.05. * *p* < 0.05; ** *p* < 0.01, * indicates that there is a significant difference between the test samples in comparison with the negative control at the effect of this concentration. The results are shown in Figure 5.

**Figure 6:**
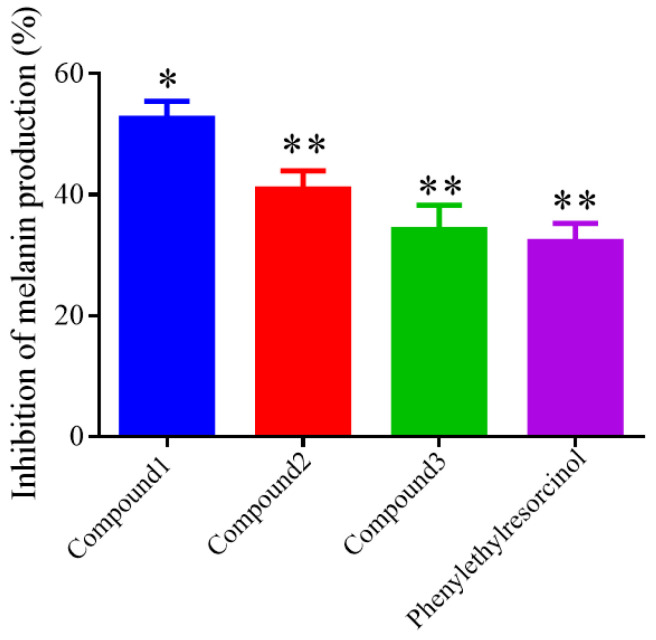
Effect of compounds **1**–**3** and the positive control phenylethylresorcinol (377) on melanin synthesis in B16 cells. * p < 0.05, ** *p* < 0.1.

As shown in Figure 5, compounds **1**–**3** and phenylethylresorcinol [18] significantly inhibited melanin synthesis in B16 cells (*p* < 0.05) with inhibition rates of 52.230%, 40.881%, 34.798% and 32.791%, respectively. The inhibitory rates of compound **1**–**3** on melanin synthesis were conspicuously higher than that of the market classic whitening ingredient phenylethylresorcinol, which indicated that compound **1**–**3** had excellent whitening efficacy (Figure 6).

**Figure 7:**
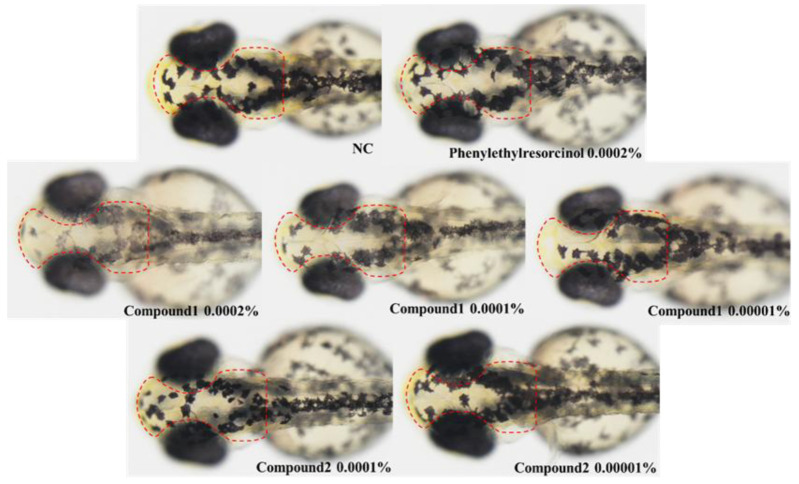
Typical diagram of whitening (red dashed areas are quantitative, NC: negative control).

As shown in Table 2 and Figure 7, both compounds **1** and **2** showed significant whitening efficacy in zebrafish. In particular, compound **1** had whitening-effect rates of 31% at a concentration of 0.0001%, and 52% at a concentration of 0.0002%. Meanwhile, compound **2** had a whitening-effect rate of 20% at a concentration of 0.0001%. Both compounds had more superior whitening efficacy than the commercially available whitening agent phenylethylresorcinol (377), which had a whitening rate of only 12% at a concentration of 0.0002% [18].

## 3. Discussion

The main aim of the present investigation was to find an active substance which (a) has a favorable skin-whitening effect (i.e., for example a potent inhibition of tyrosinase and α-glucosidase in specific cell-free or cell in vitro test systems, and on in vivo experiments inzebrafish and mice), (b) can be prepared in a highly pure form, (c) is dermatologically and toxicologically acceptable, and (d) in addition, has good stability to the effects of light. For safety, compound **1** and compound **2** did not show potential genotoxicity at all concentrations IC_50_ (22.5, 19.76 μg/mL), 1/2 IC_50_ (11.28, 9.88 μg/mL) and 1/4 IC_50_ (5.64, 4.96 μg/mL) under all of conditions of the micronucleus test. Compound **1** and compound **2** were dissolved in DMSO at concentrations of 5 mg/dish, 2.5 mg/dish, 1.6 mg/dish, 0.8 mg/dish, 0.4 mg/dish, and filtered samples were assayed with standard strains of TA97a, TA98, TA100, and TA102, with no potential genotoxicity. Compounds **1** and **2** did not show any skin phototoxicity when tested on a guinea pig. 

Our studies have shown that 1,3-bisbenzylphenylphenol derivatives fulfill the above purposes and may therefore be preferentially used as dual inhibitors of tyrosinase and α-glucosidase.

**Scheme 1:**
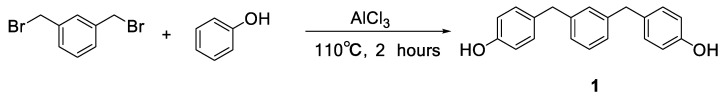
The synthesis of compound **1**.

**Scheme 2:**
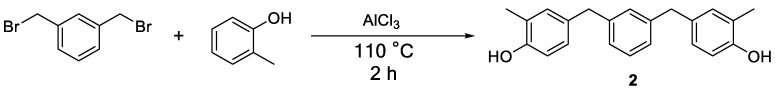
The synthesis of compound **2**.

We showed that 1,3-bisbenzylphenylphenol derivatives and, here in particular, compound **1** of Scheme 1, have more powerful tyrosinase-inhibiting activity than styrylresorcinol which is used in the cosmetic industry as a skin-lightening inhibitor (See Table 1: comparison of compounds **1**, **2** (4,4′-(1,3-phenylenebis(methylene))bis(2-methylphenol)) (Scheme 1 and Scheme 2) and styrylresorcinol). In addition, the fact that, here in particular, compounds **1** and **2** have more powerful tyrosinase-inhibiting activity compared with the known skin-lightening active compound kojic acid [8,9] was particularly surprising, and thus they can be used in particularly low—and thus toxicologically and dermatologically acceptable—concentrations in cosmetic products. In addition, compound **1** has a tyrosinase-inhibiting action that is more powerful by a factor of approximately 4 than that of kojic acid. Compound **2** has a tyrosinase-inhibiting action that is more powerful by a factor of approximately 10 than that of kojic acid [8,9]. 

The synthetic compound **1** (Scheme 1: 4,4′-(1,3-phenylenebis(methylene))diphenol) prepared in accordance with methods known from the literature confirm, for example, that compound **1** (1,3-bisbenzylphenylphenol) has a powerful tyrosinase-inhibiting, and α-glucosidase-inhibiting action, and thus are outstandingly suitable for use as skin-lightening agents and as agents for combating age spots. In this context, because of their high stability to light, they are outstandingly suitable for use as skin lighteners in cosmetic products and the like, as alternatives for or as supplements to known skin-lightening active compounds (such as, for example, hydroquinone, arbutin or ascorbic acid). The compound of Scheme 1 and the further compounds, in which the OH group is in the para-position, or OH groups are in the ortho-position with respect to one another, are, moreover, very stable to oxygen.

Tyrosinase is a crucial copper-containing enzyme involved in the production of melanin. Melasma, age spots, and freckles are examples of hyperpigmentation diseases caused by excess production of melanin. Inhibiting tyrosinase activity is a crucial method for treating these disorders along with various applications such as cosmetics, food technology, and medicine. Natural products have proven a rich source of tyrosinase inhibitors, with some molecules from plant, marine, and microbial sources showing inhibitory action [19].

α-Glucosidase inhibition can also block the modification of the sugar chains on glycoproteins from producing active tyrosinase, and there is a corresponding reduction in melanin formation [13,14]. The bioactive α-glucosidase inhibitors for the treatment of diabetes have been proven to be the most efficient remedy for controlling postprandial hyperglycemia and its detrimental physiological complications, especially in type 2 diabetes. The carbohydrate hydrolyzing enzyme, α-glucosidase, is generally competitively inhibited by the α-glucosidase inhibitors and results in the delayed glucose absorption in small intestine, ultimately controlling the postprandial hyperglycemia [20]. 

To date, no dual inhibitors of tyrosinase and α-glucosidase have been reported for use in skin-lightening agents. The compound identified in this study exhibits higher efficacy than currently known skin-lightening agents, with its mechanism potentially linked to this dual inhibition, producing a synergistic effect that enhances overall performance [11]. Interestingly, there are reports that antioxidants can exert a synergistic effect on tyrosinase inhibitory activity [18]. Compounds **1**–**3** synthesized by us exhibit antioxidant activity in addition to their tyrosinase and alpha-glucosidase inhibitory effects. This antioxidant activity may also exert a synergistic effect on their tyrosinase inhibition.

A total of 31 congeners were synthesized in this study, and these compounds were evaluated for their tyrosinase and α-glucosidase inhibitory activities, as well as their antioxidant properties. Apart from the initial compounds **1**–**3**, which exhibited outstanding activities, all subsequent synthesized compounds demonstrated weaker activities than compounds **1**–**3**. No clear structure–activity relationship was observed. Due to space constraints, the detailed activity data are omitted here. Only compounds **1** and **2** were selected as target compounds for subsequent zebrafish skin-lightening activity and safety testing.

## 4. Materials and Methods

### 4.1. General

Reversed phase silica gel (Fuji Silysia Chemical Co., Kasugai, Japan, Lichroprep RP-18, 40–70 µm) were used for column chromatography (CC). Medium-pressure liquid chromatography (MPLC) was carried out on a Biotage (Uppsala, Sweden, C-615 Pump Manager, C-605 pump module, C-660 fraction collector, RP-18 column). The analytic HPLC was performed on Zorbax SB-C18 column (5 µm, 4.6 × 150 mm, gradient elution of acetonitrile–water 10–30%, *v*/*v*, 1.0 mL/min, 0–15 min) utilizing a DAD detector on an Agilent 1260 liquid chromatography system (Agilent Technologies, Santa Clara, CA, USA). The preparative HPLC was performed on a Zorbax SB-C18 column (5 µm, 9.4 × 150 mm) utilizing a DAD detector on an Agilent 1260 liquid chromatography system (Agilent Technologies, Santa Clara, CA, USA). The Bruker Avance III 600 MHz spectrometer (Bruker, Karlsruhe, Germany) was utilized to obtain the NMR spectra. On a Q Exactive Orbitrap mass spectrometer (Thermo Scientific, Waltham, MA, USA), the HRESIMS spectra were acquired.

### 4.2. Synthesis of Active Compounds

*Synthesis of compound **1**:* 1,3-bis(bromomethyl)benzene (1.5 g, 5.73 mmol), phenol (6.2 mL, 70 mmol), aluminum trichloride (195 mg, 1.5 mmol) were sequentially weighed and heated at 110 °C under the protection of nitrogen until the disappearance of the raw materials, and the reaction was carried out for 2 h, then the reaction was cooled down to room temperature, and then the product was extracted with a saturated aqueous sodium carbonate and ethyl acetate three times, and dried with sodium sulfate, filtered and concentrated in vacuum. It was dried with sodium sulfate, filtered and spun-dried, and purified by ethyl acetate/petroleum ether (1:200, *v*/*v*) over a column to obtain the product (473.3 mg, 42% yield, white solid) (Scheme 1).

*Phenylenebis(methylene))bisphenol* (Compound **1**): white solid, ^1^H NMR (600 MHz, CD_3_OD, δ, ppm, J/Hz): 9.17 (s, 2H), 7.15 (t, *J* = 10, 1H), 7.04 (s, 1H), 6.98-6.95 (m, 6H), 6.66–6.64 (m, 4H), 3.76 (s, 4H). ^13^C NMR (150 MHz, DMSO-*d*_6_, δ, ppm): 155.9, 142.4, 131.8, 130.0, 129.3, 128.8, 126.5, 115.6, 40.7. HRMS (ESI-TOF) calcd for C_20_H_17_O_2_ [M-H]^−^ = 289.1234, found 289.1246. The ^1^H NMR spectrum and ^13^C NMR spectrum are shown in Supplementary Materials Figures S1 and S2.

*Synthesis of compound **2:*** weigh 1,3-bis(bromomethyl)benzene (0.3 g, 1.15 mmol), *o*-cresol (0.6 g, 5.75 mmol), aluminum trichloride (39 mg, 0.29 mmol), under the protection of nitrogen, 110 °C heated to the raw material disappeared, the reaction for 2 h, cooled to room temperature, direct silica gel chromatography over the column, ethyl acetate/petroleum ether (1:7.5, *v*/*v*) was used as eluent to obtain the product (40.7 mg, 26% yield, brown solid) (Scheme 2). 

*Phenylenebis(methylene))bis-2-methylphenol* (Compound **2**): brown solid, ^1^H NMR (600 MHz, DMSO-*d*_6_, *δ*, ppm, *J*/Hz): 9.05 (s, 2H), 7.15 (t, *J* = 7.6 Hz, 1H), 7.05 (s, 1H), 6.97 (d, *J* = 7.6 Hz, 2H), 6.87 (s, 2H), 6.80 (dd, *J* = 8.2, 2.3 Hz, 2H), 6.67 (d, *J* = 8.1 Hz, 2H), 3.73 (s, 4H), 2.06 (s, 6H). ^13^C NMR (150 MHz, DMSO-*d*_6_, *δ*, ppm): 153.5, 142.0, 131.3, 130.9, 128.8, 128.3, 126.7, 126.0, 123.6, 114.5, 40.4, 16.0. HRMS (QE) calcd for C_22_H_22_O_2_ [M-H]^−^ = 317.1547, found 317.1561. The ^1^H NMR spectrum and ^13^C NMR spectrum are shown in Supplementary Materials Figures S3 and S4.

**Scheme 3:**
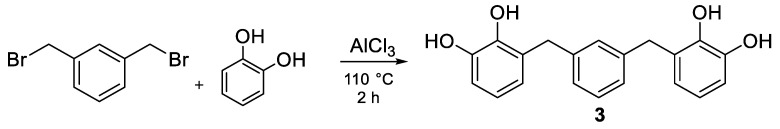
The synthesis of compound **3**.

*Synthesis of compound **3**:* 1,3-bis(bromomethyl)benzene (1.5 g, 5.73 mmol), 1,2-benzenediol (0.63 g, 5.72 mmol), aluminum trichloride (195 mg, 1.5 mmol) were weighed and heated at 110 °C under the protection of nitrogen until the disappearance of the raw materials, the reaction was carried out for 2 h. The reaction was cooled down to room temperature, and the product (124.8 mg, yield 32%, light yellow solid) could be obtained by direct silica gel chromatography on a column, and ethyl acetate/petroleum ether (1:2, *v*/*v*) as eluent. The product (124.8 mg, 32% yield, light yellow solid) was obtained with ethyl acetate/petroleum ether (1:2, *v*/*v*) as eluent (Scheme 3).

*3,3*′*-(1,3-phenylenebis(methylene))bis(benzene-1,2-diol (compound **3**)*: Light yellow solid, ^1^H NMR (500 MHz, DMSO-*d*_6_, *δ*, ppm, *J*/Hz): 8.74 (s, 2H), 8.64 (s, 2H), 7.15 (t, *J* = 7.6 Hz, 1H), 7.01 (s, 1H), 6.95 (dd, *J* = 7.6, 1.7 Hz, 2H), 6.61 (d, *J* = 8.0 Hz, 2H), 6.53 (d, *J* = 2.1 Hz, 2H), 6.44 (dd, *J* = 8.0, 2.1 Hz, 2H), 3.69 (s, 4H). ^13^C NMR (125 MHz, DMSO-*d*_6_, *δ*, ppm): 145.5, 143.9, 142.4, 132.5, 129.4, 128.7, 126.6, 119.8, 116.5, 115.9, 41.0. HRMS (QE) calcd for C_20_H_18_NaO_4_ [M+Na]^+^ = 345.1097, found 345.1097. The ^1^H NMR spectrum and ^13^C NMR spectrum are shown in Supplementary Materials Figures S5 and S6.

**Scheme 4:**
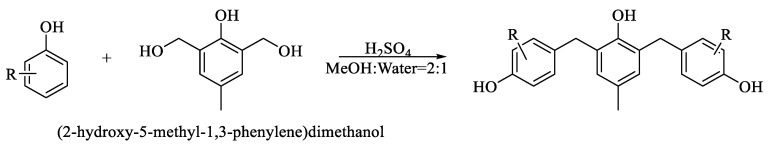
The synthesis of compound **4**–**22**.

**Figure 8:**
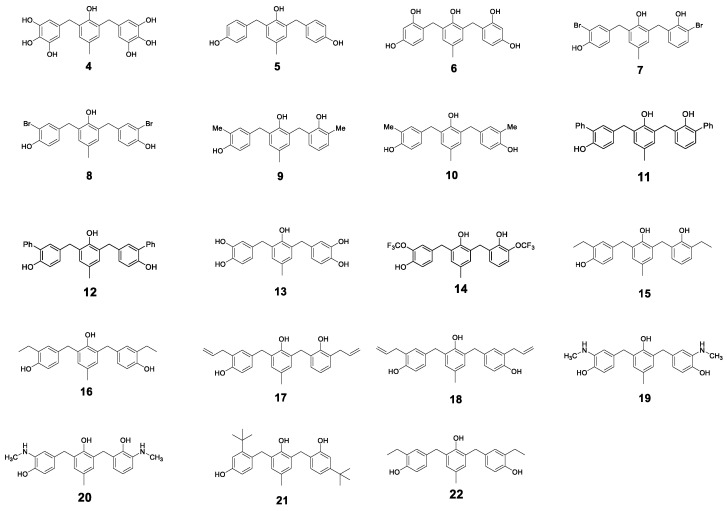
The structures of compound **4**-**22.**

*General procedures for the synthesis of compounds **4**–**22**:* To the corresponding phenol or arenes (5 equiv) in methano/H_2_O = 2:1 was added H_2_SO_4_ (catalytic amount). Then the (2-hydroxy-5-methyl-1,3-phenylene)dimethanol (1 equiv) was added. The mixture was heated at 45 °C under the protection of nitrogen until the completion of the reaction, the desired product was obtained by flash chromatography on silica gel using petroleum ether/ethyl acetate (20:1–1:1) as the eluent (Scheme 4). The structures of compound **4**–**22** see Figure 8.

*5,5*′*-((2-hydroxy-5-methyl-1,3-phenylene)bis(methylene))bis(benzene-1,2,3-triol)* (compound **4**): ^1^H NMR (500 MHz, DMSO-*d*_6_,*δ*, ppm, *J*/Hz): 8.80 (s, 3H), 8.20 (s, 4H), 6.53 (s, 2H), 6.30 (d, *J* = 8.3 Hz, 2H), 6.21 (d, *J* = 8.2 Hz, 2H), 3.68 (s, 4H), 2.02 (s, 3H); ^13^C NMR (125 MHz, DMSO-*d*_6_, *δ*, ppm): 149.7, 144.3, 143.8, 132.9, 128.1, 127.8, 127.2, 119.7, 118.6, 106.6, 29.5, 20.5. Yield: 75%.

*4,4*′*-((2-hydroxy-5-methyl-1,3-phenylene)bis(methylene) diphenol* (compound **5**): ^1^H NMR (500 MHz, DMSO-*d*_6_,*δ*, ppm, *J*/Hz): 9.23 (s, 1H), 8.15 (s, 1H), 6.98 (d, *J* = 8.5 Hz, 4H), 6.65 (d, *J* = 8.5 Hz, 4H), 6.61 (s, 2H), 3.77 (s, 4H), 2.07 (s, 3H); ^13^C NMR (125 MHz, DMSO-*d*_6_, *δ*, ppm): 155.81, 150.25, 131.66, 130.07, 129.41, 128.90, 128.16, 115.44, 35.10, 20.77. Yield: 60%.

*4,4*′*-((2-hydroxy-5-methyl-1,3-phenylene)bis(methylene))bis(benzene-1,3-diol)* (compound **6**): ^1^H NMR (500 MHz, MeOD,*δ*, ppm, *J*/Hz): 6.87 (d, *J* = 8.2 Hz, 1H), 6.72 (s, 1H), 6.31 (d, *J* = 2.5 Hz, 1H), 6.24 (dd, *J* = 8.2, 2.4 Hz, 1H), 3.75 (s, 2H), 2.12 (s, 2H). ^13^C NMR (125 MHz, MeOD, *δ*, ppm): 157.60, 155.93, 150.33, 132.00, 130.20, 129.66, 129.42, 119.99, 108.06, 103.32, 30.67, 20.72. HRMS(ESI) *m*/*z*: [M+H]^+^ Calcd for C_21_H_21_O_5_ 353.1389, found 353.1382. Yield: 52%.

*2-(3-bromo-2-hydroxybenzyl)-6-(3-bromo-4-hydroxybenzyl)-4-methylphenol* (compound **7**): ^1^H NMR (500 MHz, CDCl_3_, *δ*, ppm, *J*/Hz): 7.36 − 7.29 (m, 2H), 7.19 (dd, *J* = 7.6, 1.6 Hz, 1H), 7.07 (dd, *J* = 8.3, 2.1 Hz, 1H), 6.94 − 6.91 (m, 2H), 6.81 − 6.74 (m, 2H), 6.19 (d, *J* = 19.0 Hz, 2H), 5.43 (s, 1H), 3.92 (s, 2H), 3.86 (s, 2H), 2.22 (s, 3H). ^13^C NMR (125 MHz, CDCl_3_, *δ*, ppm): 150.45, 149.23, 148.72, 134.32, 131.87, 130.38, 130.07, 129.94, 129.84, 129.51, 128.10, 127.75, 125.98, 122.45, 115.90, 110.42, 110.17, 35.28, 31.26, 20.51. HRMS(ESI) *m*/*z*: [M+H]^+^ Calcd for C_21_H_19_Br_2_O_3_ 476.9701, found 476.9551. Yield: 37%.

*4,4*′*-((2-hydroxy-5-methyl-1,3-phenylene)bis(methylene))bis(2-bromophenol)* (compound **8**): ^1^H NMR (500 MHz, CDCl_3_, *δ*, ppm, *J*/Hz): 7.32 − 7.29 (m, 2H), 7.06 (dd, *J* = 8.2, 2.1 Hz, 2H), 6.94 (d, *J* = 8.3 Hz, 2H), 6.85 (s, 2H), 5.66 (s, 2H), 4.57 (s, 1H), 3.86 (s, 4H), 2.28 (d, *J* = 3.1 Hz, 3H). ^13^C NMR (125 MHz, CDCl_3_, *δ*, ppm): 150.65, 149.46, 133.43, 131.73, 130.06, 129.89, 129.23, 126.73, 116.11, 110.30, 35.31, 20.50. HRMS(ESI) *m*/*z*: [M+H]^+^ Calcd for C_21_H_19_Br_2_O_3_ 476.9701, found 476.9551. Yield: 26%.

*2-(2-hydroxy-3-methylbenzyl)-6-(4-hydroxy-3-methylbenzyl)-4-methylphenol* (compound **9**): ^1^H NMR (500 MHz, CDCl_3_, *δ*, ppm, *J*/Hz): 7.15 (d, *J* = 7.5 Hz, 1H), 7.03 − 6.94 (m, 3H), 6.91 (dd, *J* = 8.0 and 2.3 Hz, 1H), 6.82 (t, *J* = 4.4 Hz, 2H), 6.68 (dd, *J* = 8.1 and 1.9 Hz, 1H), 5.87 (s, 1H), 5.12 (s, 1H), 3.87 (d, *J* = 9.0 Hz, 4H), 2.25 (s, 3H), 2.22 (d, *J* = 6.1 Hz, 6H). ^13^C NMR (125 MHz, CDCl_3_, *δ*, ppm): 153.07, 151.89, 148.84, 131.66, 131.48, 131.29, 130.87, 130.14, 129.87, 129.60, 128.57, 127.54, 127.37, 126.78, 124.79, 124.73, 120.85, 115.50, 35.89, 30.66, 20.16, 15.73, 15.37. HRMS(ESI) *m*/*z*: [M-H]^−^ Calcd for C_21_H_23_O_3_ 347.1647, found 347.1669. Yield: 23%.

*4,4*′*-((2-hydroxy-5-methyl-1,3-phenylene)bis(methylene))bis(2-methylphenol)* (compound **10**): ^1^H NMR (500 MHz, CDCl_3_, *δ*, ppm, *J*/Hz): δ 6.99 (s, 2H), 6.94 − 6.87 (m, 4H), 6.65 (d, *J* = 8.1 Hz, 2H), 5.53 (s, 2H), 4.78 (s, 1H), 3.87 (s, 4H), 2.30 (s, 3H), 2.21 (s, 6H). ^13^C NMR (125 MHz, CDCl_3_, *δ*, ppm): 152.32, 149.87, 131.82, 131.15, 129.64, 129.59, 127.41, 127.08, 124.02, 115.07, 35.85, 20.55, 15.77. HRMS(ESI) *m*/*z*: [M-H]^−^ Calcd for C_23_H_23_O_3_ 347.1647, found 347.1669. Yield: 25%.

*3-(2-hydroxy-3-((6-hydroxy-[1,1*′*-biphenyl]-3-yl)methyl)-5-methylbenzyl)-[1,1*′*-biphenyl]-2-ol* (compound **11**): ^1^H NMR (600 MHz, CDCl_3_, *δ*, ppm, *J*/Hz): 7.51 − 7.46 (m, 2H), 7.46 − 7.42 (m, 6H), 7.41 − 7.35 (m, 2H), 7.29 (dd, *J* = 7.6 and 1.7 Hz, 1H), 7.14 − 7.10 (m, 3H), 7.00 (d, *J* = 2.2 Hz, 1H), 6.96 (t, *J* = 7.6 Hz, 1H), 6.89 (d, *J* = 8.9 Hz, 1H), 6.80 (d, *J* = 2.2 Hz, 1H), 6.49 (s, 1H), 6.09 (s, 1H), 5.13 (s, 1H), 3.95 (s, 2H), 3.92 (s, 2H), 2.24 (s, 3H). ^13^C NMR (150 MHz, CDCl_3_, *δ*, ppm): 151.16, 149.75, 149.26, 137.52, 137.28, 133.04, 130.74, 130.53, 130.05, 130.03, 129.72, 129.69, 129.65, 129.48, 129.43, 129.37, 128.92, 128.70, 128.35, 128.31, 128.02, 127.49, 126.84, 121.66, 116.09, 35.44, 30.72, 20.22. HRMS(ESI) *m*/*z*: [M-H]^−^ Calcd for C_33_H_27_O_3_ 471.1960, found 471.1988. Yield: 26%.

*5,5*″*-((2-hydroxy-5-methyl-1,3-phenylene)bis(methylene))bis(([1,1*′*-biphenyl]-2-ol))* (compound **12**): ^1^H NMR (500 MHz, CDCl_3_, *δ*, ppm, *J*/Hz): 7.51 − 7.35 (m, 10H), 7.13 − 7.05 (m, 4H), 6.92 − 6.85 (m, 4H), 5.22 (d, *J* = 14.6 Hz, 2H), 4.60 (s, 1H), 3.92 (s, 4H), 2.25 (s, 3H). ^13^C NMR (125 MHz, CDCl_3_, *δ*, ppm): 151.40, 150.31, 137.39, 132.39, 130.57, 130.11, 130.05, 129.50, 129.37, 128.51, 128.11, 127.45, 116.31, 35.61, 20.21. HRMS(ESI) *m*/*z*: [M-H]^−^ Calcd for C_33_H_27_O_3_ 471.1960, found 471.1988. Yield: 38%.

*4,4*′*-((2-hydroxy-5-methyl-1,3-phenylene)bis(methylene))bis(benzene-1,2-diol)* (compound **13**): ^1^H NMR (500 MHz, MeOD, *δ*, ppm, *J*/Hz): 6.68 − 6.64 (m, 4H), 6.61 (d, *J* = 2.1 Hz, 2H), 6.53 (dd, *J* = 8.0 and 2.1 Hz, 2H), 3.78 (s, 4H), 2.12 (s, 3H). ^13^C NMR (125 MHz, MeOD, *δ*, ppm): 150.97, 146.02, 144.24, 134.04, 130.19, 130.15, 129.90, 121.26, 117.10, 116.15, 36.23, 20.73. HRMS(ESI) *m*/*z*: [M-H]^−^ Calcd for C_21_H_21_O_5_ 353.1389, found 353.1381. Yield: 42%.

*2-(2-hydroxy-3-(trifluoromethoxy)benzyl)-6-(4-hydroxy-3-(trifluoromethoxy)benzyl)-4-methylphenol* (compound **14**): ^1^H NMR (500 MHz, CDCl_3_, *δ*, ppm, *J*/Hz): 7.07 − 7.04 (m, 2H), 7.01 (dd, *J* = 8.4 and 2.1 Hz, 2H), 6.95 (s, 1H), 6.93 (s, 1H), 6.83 (s, 2H), 5.41 (s, 2H), 4.39 (s, 1H), 3.87 (s, 4H), 2.25 (s, 3H). ^13^C NMR (125 MHz, CDCl_3_, *δ*, ppm): 150.00, 146.63, 136.69, 133.00, 130.51, 130.30, 128.38, 126.83, 121.80, 117.64, 35.34, 20.15. Yield: 24%.

*2-(3-ethyl-2-hydroxybenzyl)-6-(3-ethyl-4-hydroxybenzyl)-4-methylphenol* (compound **15**): ^1^H NMR (600 MHz, CDCl_3_, *δ*, ppm, *J*/Hz): 7.15 (dt, *J* = 7.6 and 1.2 Hz, 1H), 7.01 (td, *J* = 10.5, 10.0, 2.0 Hz, 3H), 6.90 (dd, *J* = 8.1and 2.3 Hz, 1H), 6.84 (ddd, *J* = 16.1, 8.1, 1.6 Hz, 2H), 6.68 (d, *J* = 8.1 Hz, 1H), 6.64 (s, 1H), 5.70 (s, 1H), 5.02 (s, 1H), 3.88 (d, *J* = 3.2 Hz, 3H), 2.61 (p, *J* = 7.4 Hz, 4H), 2.25 (s, 3H), 1.21 (td, *J* = 7.5 and 2.0 Hz, 6H). ^13^C NMR (150 MHz, CDCl_3_, *δ*, ppm): 152.72, 151.62, 148.83, 131.19, 130.94, 130.91, 130.16, 129.93, 129.83, 128.47, 127.76, 127.38, 127.31, 127.23, 126.84, 120.87, 115.81, 36.10, 30.71, 22.82, 22.69, 20.19, 13.61, 13.53. HRMS(ESI) *m*/*z*: [M+H]^+^ Calcd for C_25_H_29_O_3_ 377.2117, found 377.2112. Yield: 15%.

*4,4*′*-((2-hydroxy-5-methyl-1,3-phenylene)bis(methylene))bis(2-ethylphenol)* (compound **16**): ^1^H NMR (500 MHz, CDCl_3_, *δ*, ppm, *J*/Hz): 7.01 (d, *J* = 2.2 Hz, 2H), 6.89 (dd, *J* = 8.1 and 2.3 Hz, 2H), 6.86 (s, 2H), 6.65 (d, *J* = 8.0 Hz, 2H), 4.96 (s, 1H), 4.64 (s, 1H), 3.87 (s, 4H), 2.60 (d, *J* = 7.5 Hz, 4H), 2.26 (d, *J* = 6.6 Hz, 3H), 1.21 (t, *J* = 7.6 Hz, 6H). ^13^C NMR (150 MHz, CDCl_3_, *δ*, ppm): 151.83, 149.84, 131.91, 130.19, 129.63, 129.60, 129.51, 127.40, 126.90, 115.33, 35.92, 22.98, 20.55, 14.00. HRMS(ESI) *m*/*z*: [M+H]^+^ Calcd for C_25_H_29_O_3_ 377.2117, found 377.2112. Yield: 12%.

*2-(3-allyl-2-hydroxybenzyl)-6-(3-allyl-4-hydroxybenzyl)-4-methylphenol* (compound **17**)*:* ^1^H NMR (500 MHz, DMSO-*d_6_*, *δ*, ppm, *J*/Hz): 9.14 (s, 1H), 8.45 (d, *J* = 55.5 Hz, 2H), 6.92 − 6.87 (m, 2H), 6.85 − 6.80 (m, 2H), 6.72 − 6.65 (m, 2H), 6.65 − 6.60 (m, 2H), 6.02 − 5.86 (m, 2H), 5.09 − 4.92 (m, 4H), 3.84 (s, 2H), 3.76 (s, 2H), 3.34 (dd, *J* = 6.6 and 1.6 Hz, 2H), 3.22 (dd, *J* = 6.6 and 1.6 Hz, 2H), 2.05 (s, 3H). ^13^C NMR (125 MHz, DMSO-*d_6_*, *δ*, ppm): 152.97, 152.05, 149.66, 137.31, 137.22, 131.33, 130.17, 128.97, 128.65, 128.55, 128.20, 128.07, 127.84, 127.66, 127.36, 126.73, 125.63, 119.76, 115.49, 115.26, 114.77, 34.75, 34.21, 34.02, 30.41, 20.42. HRMS(ESI) *m*/*z*: [M+H]^+^ Calcd for C_27_H_29_O_3_ 401.2117, found 401.2115. Yield: 34%.

*4,4*′*-((2-hydroxy-5-methyl-1,3-phenylene)bis(methylene))bis(2-allylphenol)* (compound **18**): ^1^H NMR (500 MHz, CDCl_3_, *δ*, ppm, *J*/Hz): 6.98 (d, *J* = 2.3 Hz, 1H), 6.95 (dd, J = 8.2 and 2.4 Hz, 1H), 6.84 (s, 1H), 6.72 (d, *J* = 8.2 Hz, 1H), 6.05 − 5.95 (m, 1H), 5.32 (s, 1H), 5.16 (dd, *J* = 6.9 and 1.7 Hz, 2H), 5.13 (t, *J* = 1.6 Hz, 2H), 3.86 (s, 4H), 3.37 (dt, *J* = 6.4, Hz, 4H), 2.26 (s, 3H). ^13^C NMR (125 MHz, CDCl_3_, *δ*, ppm): 153.07, 136.75, 132.27, 130.88, 129.92, 128.10, 127.64, 116.53, 116.18, 35.53, 34.76, 20.17. HRMS(ESI) *m*/*z*: [M+H]^+^ Calcd for C_27_H_29_O_3_ 401.2117, found 401.2112. Yield: 27%.

*4,4*′*-((2-hydroxy-5-methyl-1,3-phenylene)bis(methylene))bis(2-(methylamino)phenol)* (compound **19**): ^1^H NMR (600 MHz, CDCl_3_, *δ*, ppm, *J*/Hz): 7.09 − 7.03 (m, 1H), 6.91 − 6.84 (m, 2H), 6.83 − 6.75 (m, 2H), 6.71 (d, *J* = 13.2 Hz, 2H), 6.56 (d, *J* = 9.1 Hz, 1H), 4.07 − 3.76 (m, 4H), 2.58 (d, *J* = 10.8 Hz, 3H), 2.28 − 2.13 (m, 6H). HRMS(ESI) *m*/*z*: [M+H]^+^ Calcd for C_23_H_27_N_2_O_3_ 379.2022, found 379.2016. Yield: 31%.

*2-(2-hydroxy-3-(methylamino)benzyl)-6-(4-hydroxy-3-(methylamino)benzyl)-4-methylphenol* (compound **20**): ^1^H NMR (600 MHz, CDCl_3_, *δ*, ppm, *J*/Hz): 6.86 (d, *J* = 2.1 Hz, 2H), 6.77 (d, *J* = 7.9 Hz, 2H), 6.73 (s, 2H), 6.58 (d, *J* = 7.9 Hz, 2H), 4.75 (s, 3H), 3.82 (s, 3H), 2.82 (s, 4H), 2.20 (s, 3H). HRMS(ESI) *m*/*z*: [M+H]^+^ Calcd for C_23_H_27_N_2_O_3_ 379.2022, found 379.2017. Yield: 28%.

*2-(4-(tert-butyl)-2-hydroxybenzyl)-6-(2-(tert-butyl)-4-hydroxybenzyl)-4-methylphenol* (compound **21**): ^1^H NMR (600 MHz, MeOD, *δ*, ppm, *J*/Hz): 7.05 (d, *J* = 7.9 Hz, 1H), 6.90 (d, *J* = 2.6 Hz, 1H), 6.86 (d, *J* = 1.9 Hz, 1H), 6.82 − 6.79 (m, 2H), 6.76 (d, *J* = 8.3 Hz, 1H), 6.54 (dd, *J* = 8.3 and 2.6 Hz, 1H), 6.28 (d, *J* = 2.2 Hz, 1H), 4.09 (s, 2H), 3.82 (s, 2H), 2.04 (s, 3H), 1.33 (s, 9H), 1.25 (s, 9H). ^13^C NMR (150 MHz, MeOD, *δ*, ppm): 155.99, 154.17, 151.85, 150.67, 150.63, 135.06, 131.26, 131.11, 130.39, 129.81, 129.63, 129.40, 128.50, 125.66, 118.36, 114.27, 113.41, 112.99, 36.51, 35.14, 35.00, 31.97, 31.79, 31.22, 20.82. HRMS(ESI) *m*/*z*: [M+H]^+^ Calcd for C_29_H_37_O_3_ 433.2743, found 377.2736. Yield: 23%.

*4,4*′*-((2-hydroxy-5-methyl-1,3-phenylene)bis(methylene))bis(2-ethylphenol)* (compound **22**): ^1^H NMR (600 MHz, CDCl_3_, *δ*, ppm, *J*/Hz): 7.01 (d, *J* = 2.2 Hz, 2H), 6.89 (dd, *J* = 8.1 and 2.3 Hz, 2H), 6.86 (s, 2H), 6.65 (d, *J* = 8.0 Hz, 2H), 4.96 (s, 1H), 4.64 (s, 1H), 3.87 (s, 4H), 2.60 (d, *J* = 7.5 Hz, 4H), 2.26 (d, *J* = 6.6 Hz, 3H), 1.21 (t, *J* = 7.6 Hz, 6H). ^13^C NMR (150 MHz, CDCl_3_, *δ*, ppm): 151.83, 149.84, 131.91, 130.19, 129.63, 129.60, 129.51, 127.40, 126.90, 115.33, 35.92, 22.98, 20.55, 14.00. HRMS(ESI) *m*/*z*: [M+H]^+^ Calcd for C_25_H_29_O_3_ 377.2117, found 377.2112. Yield: 33%.

**Scheme 5:**
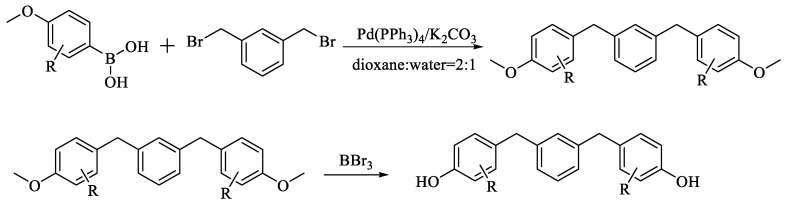
The synthesis of compound **23**-**31**.

**Figure 9:**
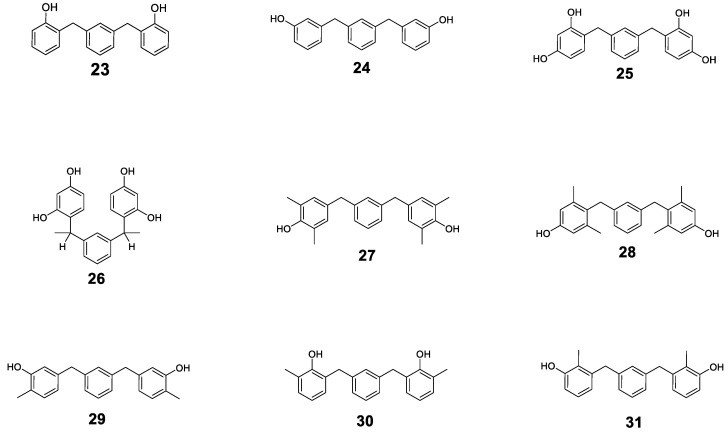
The structures of compound **23**-**31.**

*General procedures for the synthesis of **23**-**31**:* To the solution of phenylboronic acid (2.4 equiv) and 1,3-bis(bromomethyl)benzene in Dioxane/H_2_O = 2:1 was added K_2_CO_3_ (4.8 equiv) under nitrogen atmosphere. Then the mixture was heated at 100 °C until the completion of the reaction, the desired product was obtained by flash chromatography on silica gel using petroleum ether/ethyl acetate (20:1-1:1) as the eluent. The obtained coupling product was dissolved in DCM. To the above solution (1 equiv) was added BBr_3_ (1M, 4.8 eq) dropwise. After completion of the reaction by TLC analysis, the reaction was quenched and extracted by DCM for 3 times. The combined organic layer was washed, dried, concentrated and purified by flash chromatography on silica gel to give the product (Scheme 5). The structures of compound **23**-**31** see Figure 9.

*2,2*′*-(1,3-phenylenebis(methylene))diphenol* (compound **23**): ^1^H NMR (600 MHz, CDCl_3_, *δ*, ppm, *J*/Hz): 7.23 (t, *J* = 7.6 Hz, 1H), 7.18 (s, 1H), 7.17–7.11 (m, 4H), 7.08 (d, *J* = 7.8 Hz, 2H), 6.94–6.89 (m, 2H), 6.77 (d, *J* = 7.8 Hz, 2H), 5.10 (s, 2H), 3.98 (s, 4H). ^13^C NMR (150 MHz, CDCl_3_, *δ*, ppm): 153.59, 140.16, 130.88, 129.14, 128.89, 127.75, 126.97, 126.63, 120.87, 115.68, 36.25. Yield: 76%.

*3,3*′*-(1,3-phenylenebis(methylene))diphenol* (compound **24**): ^1^H NMR (600 MHz, CDCl_3_, *δ*, ppm, *J*/Hz): 7.19 (t, *J* = 7.6 Hz, 1H), 7.14 (t, *J* = 7.8 Hz, 2H), 7.04 (s, 1H), 7.02 (dd, *J* = 7.5 and 1.8 Hz, 2H), 6.78 (d, *J* = 7.5 Hz, 2H), 6.68 (dd, *J* = 8.2 and 2.3 Hz, 2H), 6.61 (t, *J* = 2.0 Hz, 2H), 6.22 (s, 2H), 3.88 (s, 4H). ^13^C NMR (150 MHz, CDCl_3_, *δ*, ppm): 171.94, 155.66, 143.02, 140.80, 129.77, 129.50, 128.57, 126.79, 121.14, 115.74, 113.03, 41.51. Yield: 72%.

*4,4*′*-(1,3-phenylenebis(methylene))bis(benzene-1,3-diol)* (compound **25**): ^1^H NMR (500 MHz, DMSO-d_6_, *δ*, ppm, *J*/Hz): 9.15 (s, 2H), 8.99 (s, 2H), 7.08 (t, *J* = 7.5 Hz, 1H), 7.04 (d, *J* = 2.1 Hz, 1H), 6.90 (dd, *J* = 7.5, 1.9 Hz, 2H), 6.73 (dd, *J* = 8.2, 1.7 Hz, 2H), 6.27 (s, 2H), 6.11 (dt, *J* = 8.2, 2.2 Hz, 2H), 3.67 (s, 4H); ^13^C NMR (125 MHz, CDCl_3_, *δ*, ppm): 156.5, 155.6, 141.7, 130.6, 128.9, 127.8, 125.7, 118.0, 106.0, 102.4, 34.6. Yield: 57%.

*4,4*′*-(1,3-phenylenebis(ethane-1,1-diyl))bis(benzene-1,3-diol)* (compound **26**): ^1^H NMR (500 MHz, DMSO-*d_6_*, *δ*, ppm, *J*/Hz): 9.11 (d, *J* = 5.0 Hz, 1H), 8.96 (s, 1H), 7.15–7.04 (m, 1H), 6.93 (d, *J* = 7.5 Hz, 1H), 6.79 (dd, *J* = 8.4, 3.9 Hz, 1H), 6.23 (s, 1H), 6.13 (d, *J* = 8.3 Hz, 1H), 4.31–4.22 (m, 1H), 3.35 (s, 3H), 1.40 (d, *J* = 7.6 Hz, 3H); ^13^C NMR (150 MHz, DMSO-*d_6_*, *δ*, ppm): 156.53, 156.52, 155.46, 155.44, 146.93, 146.88, 128.15, 128.01, 127.96, 127.37, 127.07, 124.93, 124.78, 123.69, 123.61, 106.43, 106.40, 102.75, 36.75, 36.70, 21.69, 21.62. Yield: 62%.

*4,4*′*-(1,3-phenylenebis(methylene))bis(2,6-dimethylphenol)* (compound **27**): ^1^H NMR (600 MHz, DMSO-*d_6_*, *δ*, ppm, *J*/Hz): 8.01 (s, 2H), 7.14 (t, *J* = 7.6 Hz, 1H), 7.05 (s, 1H), 6.96 (dd, *J* = 7.6, 1.3 Hz, 2H), 6.73 (s, 4H), 3.70 (s, 4H), 2.11 (s, 12H); ^13^C NMR (150 MHz, DMSO-*d_6_*, *δ*, ppm): 151.76, 142.46, 132.08, 129.32, 128.88, 128.73, 126.47, 124.50, 40.89, 17.12. Yield: 78%.

*4,4*′*-(1,3-phenylenebis(methylene))bis(3,5-dimethylphenol)* (compound **28**): ^1^H NMR (500 MHz, DMSO-*d_6_*,*δ*, ppm, *J*/Hz): 9.01 (s, 1H), 7.06 (t, *J* = 7.6 Hz, 1H), 6.76 (s, 1H), 6.70 (d, *J* = 7.5 Hz, 1H), 6.45 (s, 2H), 3.79 (s, 2H), 2.06 (s, 6H); ^13^C NMR (125 MHz, DMSO-*d_6_*) δ 155.59, 140.82, 137.90, 128.67, 127.98, 127.76, 125.23, 115.23, 34.14, 20.45. Yield: 82%.

*5,5*′*-(1,3-phenylenebis(methylene))bis(2-methylphenol)* (compound **29**): ^1^H NMR (600 MHz, DMSO-*d_6_*,*δ*, ppm, *J*/Hz): 9.13 (s, 2H), 7.18 (t, *J* = 7.5 Hz, 1H), 7.05 (s, 1H), 6.99 (d, *J* = 7.3 Hz, 2H), 6.94 (d, *J* = 7.4 Hz, 2H), 6.63 − 6.51 (m, 4H), 3.76 (s, 4H), 2.06 (s, 6H); ^13^C NMR (150 MHz, DMSO-*d_6_*, *δ*, ppm): 155.74, 141.96, 140.13, 130.90, 129.53, 128.81, 126.77, 121.79, 119.68, 115.38, 41.32, 16.11. Yield: 89%.

*6,6*′*-(1,3-phenylenebis(methylene))bis(2-methylphenol)* (compound **30**): ^1^H NMR (500 MHz, DMSO-*d_6_*,*δ*, ppm, *J*/Hz): 8.27 (s, 2H), 7.18 − 7.09 (m, 2H), 6.95 (d, *J* = 7.4 Hz, 2H), 6.91 (d, *J* = 6.9 Hz, 2H), 6.83 (d, *J* = 7.4 Hz, 2H), 6.65 (t, *J* = 7.4 Hz, 2H), 3.86 (s, 4H), 2.16 (s, 6H); ^13^C NMR (126 MHz, DMSO-*d_6_*, *δ*, ppm): 153.18, 141.48, 129.72, 129.09, 128.47, 128.46, 128.41, 126.52, 124.96, 119.78. Yield: 74%.

*3,3*′*-(1,3-phenylenebis(methylene))bis(2-methylphenol)* (compound **31**): ^1^H NMR (600 MHz, DMSO-*d_6_*,*δ*, ppm, *J*/Hz): 9.21 (s, 2H), 7.14 (t, *J* = 7.6 Hz, 1H), 6.98 (d, *J* = 12.8 Hz, 1H), 6.95–6.87 (m, 4H), 6.69 (d, *J* = 7.9 Hz, 2H), 6.57 (d, *J* = 7.4 Hz, 2H), 3.85 (s, 4H), 1.99 (s, 6H); ^13^C NMR (151 MHz, DMSO-*d_6_*, *δ*, ppm): 155.84, 141.03, 140.69, 129.33, 128.69, 126.40, 126.25, 122.78, 121.11, 113.27, 39.41, 11.95. Yield: 85%.

### 4.3. Inhibition of Tyrosinase Activity by Compounds **1**–**3**

*Solution preparation:* Phosphate buffer (0.2 M, pH 6.8): weigh Na_2_HPO_4_ 2.84 g, NaH_2_PO_4_ 2.4 g, respectively, dissolved in purified water and fixed to 100 mL, mixed 1:1 equal volume and adjusted pH to 6.8. L-dopa solution: weigh L-dopa 3.95 mg, dissolve in 10 mL phosphate buffer to make 2 mM L-dopa solution. Tyrosinase solution: weigh 1 mg of tyrosinase (enzyme activity of 500 U/mg) and dissolve it in 5 mL of pH 6.8 phosphate buffer to make tyrosinase solution with enzyme activity of 100 U/mL.

Sample working solution: precision weighing of compounds **1**–**3**, kojic acid 10 mg each placed in 500 μL ultrapure water, followed by a slow dropwise addition of 1 M NaOH until the solution is clear and transparent, and finally made up to 1 mL with purified water to formulate a 10 mg/mL mother solution; then phosphate buffer will be tested compounds and the positive control kojic acid diluted to a concentration of 31.2, 62.5, 125, 250, and 500 μg/mL of sample working solution.

### 4.4. Tyrosinase Activity Assay

A standard protocol with minor changes was followed to evaluate the mushroom tyrosinase inhibitory activity of the test compounds [21]. Four experimental groups were set up: sample group A1, sample negative control group A2, enzyme standard group B1 and enzyme negative control group B2; three parallels were set up for each sample, and 200 μL reaction system was prepared according to Table 1. The corresponding volume of phosphate buffer, each concentration of the samples to be tested (final concentrations of 3.12, 6.25, 12.5, 25 and 50 μg/mL), tyrosinase solution (final concentration of 20 U/mL) and the reaction substrate L-dopa (final concentration of 1.2 mM) were added to the 96-well plate in turn, and the reaction system was incubated in the microtiter plate thermostat at 37 °C for 30 min, followed by incubation in the multifunctional enzyme marker system for 30 min, followed by the determination of absorbance at 475 nm in a microplate reader (SPARK 10M, TECAN, Männedorf, Switzerland) for each test group.

The inhibition of tyrosinase by the compounds to be tested was calculated as follows:

Tyrosinase inhibition (%) = [1 − (A1 − A2)/(B1 − B2)] × 100%

### 4.5. Studies on the Inhibition of α-Glucosidase Activity by Compounds **1**–**3**

*Solution preparation:* Phosphate buffer (0.2 M, pH 6.8): weigh Na_2_HPO_4_ 2.84 g, NaH_2_PO_4_ 2.4 g, respectively, with purified water dissolved and fixed to 100 mL, 1:1 equal volume mixing and adjust the pH to 6.8.

α-Glucosidase solution: α-glucosidase powder from brewer’s yeast was prepared into 1 U/mL α-glucosidase solution with phosphate buffer (0.2 M, pH 6.8), and stored at −20 °C.

Substrate PNPG solution: 211 mg of 4-nitrophenyl-α-D-glucopyranoside (PNPG) was weighed precisely, and added to 70 mL of phosphate buffer to dissolve homogeneously, and then prepared into a substrate storage solution of 10 mM, and stored at −20 °C away from light.

Positive control working solution: acarbose was selected as the positive control in this experiment, 103.3 mg of acarbose powder was weighed precisely, and 1 mL of phosphate buffer was used to dissolve and mix well to form a 160 mM acarbose masterbatch. The master mix was then diluted to 2.5, 5, 10, 20, 40, 80 and 160 mM with phosphate buffer to form the positive control working solution.

Compound working solutions: 2.90 mg of compound **1**, 3.19 mg of compound **2** and 3.23 mg of compound **3** were weighed precisely and placed in 500 mL of ultrapure water, 1 M NaOH was added slowly until the solution was clear and transparent, and finally made up to 1 mL with purified water to form a 10 mM masterbatch of the compounds. It was then diluted with phosphate buffer to working solutions of compound **1** at concentrations of 0.062, 0.125, 0.25, 0.5, 1 and 2 mM, compound **2** at concentrations of 0.025, 0.05, 0.1, 0.2, 0.4, and 0.8 mM, and compound **3** at concentrations of 3.12, 6.25, 12.5, 25, 50 and 100 μM.

*α-Glucosidase activity assay:* The inhibitory activity of compounds **1**–**3** against α-glucosidase and their inhibition mechanism of action were investigated according to the method with a slight modification [22]. Four test groups were set up: blank group A, enzyme standard group B, sample negative control group C and sample group D. Three parallels were set up for each sample. 600 μL of reaction system was prepared, and the corresponding volume of phosphate buffer, the working solution of each concentration of the samples to be tested (the final concentration was 20-fold dilution of the working solution of the test compounds), the α-glucosidase solution (the final concentration of 0.005 U/mL), and the substrate of the reaction, PNPG (the final concentration of 0.5 mM) were added to the 96-well plate, and then mixed thoroughly. The reaction system was incubated at 37 °C for 50 min, and then the reaction was terminated by adding 60 μL of 0.1 mol/L Na_2_CO_3_ solution. The absorbance at 405 nm of each test group was measured by placing the 96-well plate to be tested in a microplate reader (SPARK 10M, TECAN). α-Glucosidase inhibition was calculated by the following formula:

α-Glucosidase inhibition rate (%) = [1 − (D − C)/(B − A)] × 100%

### 4.6. Antioxidant Activity of Compounds **1**–**3**

The ABTS assay was conducted as described [23]. The ABTS radical stock solution was prepared by mixing 7.4 mmol/L ABTS solution and 2.6 mmol/L K_2_S_2_O_8_ solution in equal volume and standing at room temperature for 16 h, protected from light. The ABTS radical stock solution was diluted with phosphate-buffered solution (pH 7.4, 10 mmol/L) to achieve an absorbance of 0.70 ± 0.05 at 734 nm, and the ABTS working solution was made. 200 μL of ABTS working solution was mixed with 10 μL of phosphate-buffered solution (pH 7.4, 10 mmol/L), and the absorbance at 734 nm was set as A0. 10 mg of compound **1** was weighed precisely and dissolved in 500 μL of purified water, and 1 mol/L NaOH was added dropwise until the solution was clarified and transparent, and then it was finally made up with purified water to 1 mL. The solution was made up to 1 mL with purified water, so that it was formulated as 10 mg/mL mother liquor. Then dilute the mother liquor to be tested with phosphate-buffered solution (pH 7.4, 10 mmol/L) to the corresponding concentration. The positive control drug, Trolox (water-soluble vitamin E), was prepared directly in pure water as a 10 mg/mL mix and then diluted with water to the appropriate concentration. 10 μL of sample solution of different concentrations were mixed with 200 μL of ABTS working solution to give final concentrations of 0.9, 1.79, 3.58, 7.15, 10.73, 14.30, 25 and 50 μg/mL for the positive drug Vitamin E, and final concentrations of 0.62, 1.25, 2.50, 5.00, 10, 20, 40 and 50 μg/mL for compound **1**, and the final concentrations of compounds **2** and **3** were 0.78, 1.56, 3.12, 6.25, 12.5, 25 and 50 μg/mL. The mixed sample solutions were allowed to stand at room temperature for 10 min, and the absorbance (Ai) was measured at a wavelength of 734 nm. Meanwhile, 10 μL of the corresponding concentration of the sample solution was mixed with 200 μL of 10 mmol/L phosphate-buffered solution of pH 7.4, and its background absorbance at the wavelength of 734 nm was measured Aj. Three parallels were set for each sample concentration. The formula for calculating the scavenging rate of samples for ABTS radicals was as follows:

ABTS radical scavenging rate (%) = [1 − (Ai − Aj)/A0] × 100%

### 4.7. Compounds **1**–**3** Cellular Activity Assay

Treatment of test samples: compounds **1**–**3**, phenylethylresorcinol were dissolved in DMSO and then diluted with 1640 culture medium to form a masterbatch with a concentration of 38.6 μmol/L, and then diluted the masterbatch with culture medium to form a series of samples with concentrations of 19.3, 9.65, 4.825, and 2.413 μmol/L for backup. 1640 Basal medium is used as negative control group [24].

Cell activity test: B16 cells were seeded into 96-well plates and incubated for 24 h. Then the cells were incubated in 1640 basal medium containing different concentrations of test samples for another 24 h. OD490 nm was detected by MTT assay, and the effect of the test samples on B16 cell viability was analyzed by *t*-test.

### 4.8. Compounds **1**–**3** Cellular Melanin Synthesis Inhibition Assay

Melanin synthesis inhibition in the cells was determined using spectrophotometry referred to those reported [25]. The 19.3 μmol/L compounds **1**–**3**, phenylethylresorcinol were used as the experimental sample, and 1640 basal medium was used as the negative control. B16 cells were seeded into 6-well plates and incubated for 24 h. Then the cells were incubated in 1640 basal medium containing different concentrations of test samples for 48 h. After incubation, the B16 cells in each well were washed twice with PBS, trypsinization under 200 μL 0.25% pancreatic enzymes, and collected into a centrifuge tube for 5 min centrifugation. Subsequently, 200 μL of 1 M NaOH containing 10% DMSO were added to each tube for thorough cell lysis under even shaking. Finally, the lysis was transferred into the 96-well plate and the absorbance at 405 nm was detected by a microplate reader. The inhibition rate of melanin synthesis was calculated.

Melanin production inhibition rate (%) = (C − T)/C × 100

where

T—the absorbance of the test samples;

C—three times average absorbance of negative control group.

### 4.9. Zebrafish Whitening Efficacy Tests

Zebrafish are transparent in the early stages of development, and melanin begins to grow from the retinal epithelium at 24 h of embryonic development. Pigment cells originate from neural crest cells, a group of cells differentiated from dorsal ectoderm, and then proliferate, migrate and differentiate into pigment mother cells. Intervening in the process of melanin formation can inhibit melanin formation. The whiteness of the zebrafish skin was used to evaluate the whitening effect of the samples [26].

Wild-type AB strain zebrafish was used for the test system. The age of zebrafish used was 6 hpf (6 h post fertilization). Sample size per group is 15 fish (*n*= 15). Adults were reared and bred according to the standard rearing and breeding methods of our laboratory, and in accordance with the requirements of the international AAALAC accreditation.

Zebrafish were randomly selected in 6-well plates with 15 fish per well. The samples were prepared into a suspension that could be dispersed uniformly in water, and a normal control group was set up at the same time, with a volume of 3 mL per well, and the samples were incubated at 28 °C and protected from light for 45 h. Ten randomly selected zebrafish in each experimental group were photographed under a dissecting microscope, and the data were analyzed and collected by using advanced image-processing software, to analyze the intensity of the melanin signals (S) on the head of the zebrafish, and then the formulae were used to calculate and judge whether the samples had the whitening effect or not. The data were analyzed to determine whether the samples had whitening effect according to the formula (Table 2, Figure 7).

Whitening effect (%) = S (Normal control group) − S (Sample group)/S (Normal control group) × 100%

### 4.10. In Vitro Mammalian Cell Micronucleus Test

The in vitro micronucleus (MNvit) test is a genotoxicity test for the detection of micronuclei (MN) in the cytoplasm of interphase cells [27,28]. Micronuclei may originate from acentric chromosome fragments (i.e., lacking a centromere), or whole chromosomes that are unable to migrate to the poles during the anaphase stage of cell division. Therefore, the MNvit test is an in vitro method that provides a comprehensive basis for investigating chromosome damaging potential in vitro because both aneugens and clastogens can be detected in cells that have undergone cell division during or after exposure to the test chemical. Micronuclei represent damage that has been transmitted to daughter cells, whereas chromosome aberrations scored in metaphase cells may not be transmitted. In either case, the changes may not be compatible with cell survival.

DMSO was used as the solvent, and a certain amount of sample was weighed and dissolved. The final concentrations of the samples in the medium are IC_50_, 1/2 IC_50_ and 1/4 IC_50_ respectively. Mouse lymphoma cells L5178Y were used. The proportion of the medium is 10% fetal bovine serum, 90% DMEM high glucose medium and 1% antibiotics. The medium is made of 10% fetal bovine serum, 90% DMEM high sugar medium and 1% antibiotics. Cultivation temperature: 37 °C; CO_2_ concentration: 5%. Prepare cell suspension using cells with not more than 32 passages, plant the prepared cell suspension into 24-well cell culture plates, the planting volume is 1mL/well, and the number of cells in each well is 4 × 10^5^ cells/well. The cells were incubated in a carbon dioxide incubator at 37 °C with 5% CO_2_ for about 24 h. In the corresponding wells of the plates, 1 mL of prepared solvent control solution containing cells and medium, positive control solution, and different doses of test samples were added respectively; the cells in the plates were exposed in three ways, including: short-term exposure without metabolic activation system (S9 mixture), addition of metabolic activation system (S9 mixture), short-term exposure, short-term exposure with metabolic activation system (S9 mixture), and short-term exposure with metabolic activation system (S9 mixture). The cells in the cell culture plates were exposed in three ways, including: short-term exposure without metabolic activation system (S9 mix), short-term exposure with metabolic activation system (S9 mix), and long-term exposure without metabolic activation system (S9 mix). The plates were incubated in a carbon dioxide incubator according to the requirements of different groups. After incubation, collect the cell suspension from the plates in a centrifuge tube and subject it to hypotonic treatment. Drop the fixed cell suspension onto a slide, one slide for each culture well. After staining the prepared micronucleus slides, observe 1000 cells under the microscope and record the number of micronucleus cells.

Positive Result Determination: Micronuclei rate showed a statistically significant increase (*p*-value < 0.05 considered significant) in at least one of the test doses compared to the concurrent solvent control; the increase in micronuclei rate was dose-dependent; none of the test results at any of the test doses were within the distribution of the historical negative control data (within the 95% range).

Determination of Negative Results: Micronuclei rate did not show a statistically significant increase at any of the doses tested compared to the concurrent solvent control. Micronuclei rate was not dose dependent. All test results were within the range (95%) of the historical solvent control data.

### 4.11. Bacterial Reverse Mutation Test

The bacterial reverse mutation test uses amino acid-requiring strains of *Salmonella typhimurium* and *Escherichia coli* to detect point mutations, which involve substitution, addition or deletion of one or a few DNA base pairs. The principle of this bacterial reverse mutation test is to detect mutations that revert the mutations present in the test strains, and restore the functional capability of the bacteria for the synthesis of an essential amino acid. The revertant bacteria are detected by their ability to grow in the absence of the amino acid required by the parent test strain [29].

Point mutations are the cause of many human genetic diseases and there is substantial evidence that point mutations in oncogenes and tumor suppressor genes of somatic cells are involved in tumor formation in humans and experimental animals. The bacterial reverse mutation test is rapid, inexpensive and relatively easy to perform. Many of the test strains have several features that make them more sensitive for the detection of mutations, including responsive DNA sequences at the reversion sites, increased cell permeability to large molecules and elimination of DNA repair systems or enhancement of error-prone DNA repair processes. The specificity of the test strains can provide some useful information on the types of mutations that are induced by genotoxic agents. A very large database of results for a wide variety of structures is available for bacterial reverse mutation tests and well-established methodologies have been developed for testing chemicals with different physico-chemical properties, including volatile compounds.

The samples were dissolved in DMSO at concentrations of 5 mg/dish, 2.5 mg/dish, 1.6 mg/dish, 0.8 mg/dish, 0.4 mg/dish, filtered and made into samples for use. *Salmonella typhimurium* TA97a, TA98, TA100, TA102 strains were used. Take appropriate amount of nutrient broth medium, add it to the test tube, inoculate the strains of the main plate into the nutrient broth medium, and incubate it at 37 °C, 150 rpm under the condition of oscillation for 10 h, and the number of viable bacteria in each ml of the culture solution should not be less than 1 × 10^9^/mL, and then determine the OD_650_.

Prepare the bottom layer medium and top layer agar: add 0.1 mL of fresh bacterial solution of the test strain, the test material (according to the set concentration of the test material to determine the specific amount of the test material), and 0.5 mL of 10% S9 mixture to 2 mL of insulated top layer medium, shake and mix well, and then pour it into the bottom layer medium quickly, rotate the dish to make the top layer medium evenly distributed in the bottom layer medium, and then put it on the flat surface to solidify. The test material is taken as 5 mg/dish, 2.5 mg/dish, 1.6 mg/dish, 0.8 mg/dish, 0.4 mg/dish, and three parallels are set for each concentration. Positive control, solvent control and blank control are also required with 3 parallels for each group. The solidified plates were incubated in an incubator at 37 °C for 48 h, the results were observed and the number of colonies was recorded.

Determination of Positive Results: Directly count the number of revertant mutant colonies grown on the medium, if the number of revertant mutant colonies of TA97a, TA98, TA100, TA102 of the test material should increase by more than one fold (i.e., the number of revertant mutant colonies is equal to or greater than that of the solvent control by more than two fold) under the condition of good background growth, and there is a dose–response relationship, it can be regarded as a positive result of the mutation of the test material. The mutagenicity test is considered positive. If there is a significant difference in the number of revertant mutant colonies in at least one or more strains at least one concentration of the test material, but there is no dose–response relationship, if the data are reproducible and there is a statistically significant positive response, then the mutation assay of the test material can be considered positive.

Determination of negative results: If the data are repeatable and statistically significant, it can be regarded as a positive result for the mutation test of the test article if the positive reaction is not consistent with the above positive reactions. Under the condition of any strain or with or without the addition of the S9 activation system, and at any concentration of the test article, there is no significant increase in the number of mutant colonies of the test article as compared with that of spontaneous mutation and there is no dose–response relationship, so the test article can be regarded as a negative result of the mutation test, and the test article does not induce mutation to the tested strains under the conditions of the test article.

### 4.12. Skin Phototoxicity Test

Before the skin phototoxicity tester was used, the light intensity (mW/cm^2^) was measured at 6 points in the back irradiation area of the experimental animal (Hartley Guinea pigs, 300–350 g, 6 in the test group, half female and half male), and the average value was calculated. Under the experimental conditions, the average light intensity is about 7.011 mW/cm^2^. The irradiation dose was 10 J/cm^2^ and was calculated according to the following formula: Irradiation time (sec) = Irradiation dose (10,000 mJ/cm^2^)/Light intensity (mJ/cm^2^/s)

Under the conditions of this experiment, the irradiation time was 1426 s. The animals were acclimatized in the experimental animal room environment for 3 days before the test. 24 h before the formal phototoxicity test, the skin on both sides of the animal spine was dehaired, and the skin of the test site was intact, without damage or abnormality according to the “cosmetics safety technical specification” (2015 edition), four dehair areas were prepared each with an area of about 2 × 2 cm.

The animal was fixed, and 0.2 mL of sample was applied to the dehaired areas 1 and 2 of the animal as shown in specification. After 30 min, the left side (dehaired areas 1 and 3) was covered with aluminum foil, fixed with adhesive tape, and the right side (dehaired areas 2 and 4) was irradiated with UVA. The skin reaction was observed at 1, 24, 48 and 72h after the end, and the skin reaction score of each animal was determined according to the standard. The sample is judged to be phototoxic when the number of animals with the sum of skin reaction scores of 2 or more in the irradiated area after the sample is coated and no skin reaction occurs in the unirradiated area. The test substances and positive control (8-methoxy psoralene) were diluted to 20% concentration with peanut oil [30].

## 5. Conclusions

The diarylheptene polyphenolic compound, previously isolated from an aquatic plant of edible vegetables of the Bai ethnic group in Yunnan *Ottelia acuminata var acuminata* with significant α-glucosidase inhibitory activity, was used as a natural template structure of the lead compound. We simplify and replace their open-chain flexible molecular structures into rigid benzene ring polyphenolic structural analogs by using conformational restriction and bioisosterism strategies (Figure 1). A series of 1,3-bis-benzylbenzil phenolic compounds were designed and synthesized, and their tyrosinase, α-glucosidase inhibitory activities, antioxidant activities and cellular melanogenesis inhibitory activities were investigated. It was found that these compounds showed potent antioxidant, tyrosinase, and α-glucosidase inhibitory activities. Compounds **1** and **2** were selected for further evaluation of their whitening effect and safety. Compounds **1** and **2** was found to inhibit the two target enzymes (tyrosinase and α-glucosidase) engaged in skin-whitening and aging with comparable IC_50_ values to the reference drugs. They showed potent whitening efficacy in zebrafish. Especially for compound 1, it had whitening-effect rates of 31% at a concentration of 0.0001%, and 52% at a concentration of 0.0002%. Both compounds had more superior whitening efficacy than commercially available whitening agent phenylethylresorcinol (377), which was used as a positive control. Compounds **1** and **2** did not show any genotoxicity and skin phototoxicity at the test concentration levels.

In summary, compounds **1** and **2** exhibit promising prospects for development as a novel cosmetic whitening agent, both in terms of efficacy and safety. Of particular significance is that these two compounds show tyrosinase inhibition, α-glucosidase inhibition, and antioxidant activity. This multi-faceted mechanism of action represents a novel development strategy, contributing to synergistic whitening effects.

## 6. Patents

The research work described herein has been submitted for patent application.

## Figures and Tables

**Table 1:** IC_50_ values of the relevant activities of the three compounds.

	Tyrosinase IC_50_ (μg/mL)	α-Glucosidase IC_50_ (μM)	Antioxidant IC_50_ (μg/mL)
Compound **1**	10.43	21.77	6.78
Compound **2**	4.36	8.91	2.56
Compound **3**	>50	0.95	2.10
Positive Control	Kojic acid 18.61	Acarbose 481.7	Vitamin E 4.75

**Table 2:** Test results.

	Test Concentration (m/m)	Whiten Effect	*p*
Compound **1**	0.0002%	52%	<0.001
	0.0001%	31%	<0.01
	0.00001%	15%	>0.05
Compound **2**	0.0001%	20%	<0.001
	0.00001%	5%	>0.05
Phenylethylresorcinol	0.0002%	12%	<0.01

## Data Availability

The data that support the findings of this study are available from the corresponding author upon reasonable request.

## Supplementary Materials

The following supporting information can be downloaded at https://www.mdpi.com/article/10.3390/molecules31030573/s1, Figure S1: The ^1^H NMR Spectra of compound **1**; Figure S2. The ^13^C NMR spectra of compound **1**; Figure S3. The ^1^H NMR spectra of compound **2**; Figure S4. The ^13^C NMR spectra of compound **2**; Figure S5. The ^1^H NMR spectra of compound **3**; Figure S6. The 13C NMR spectra of compound **3**.
