# Supplementary material for: Novel Tyrosinase and α-Glucosidase Inhibitors: 1,3-Bisbenzylphenylphenol and Congeners as Cosmetic Whitening Agents Based on Natural Products
Source: Molecules. 2026 Feb 6;31(3):573. doi: 10.3390/molecules31030573 (PMC12899787; doi:10.3390/molecules31030573)
Supplement: Supplementary file 1 [file molecules-31-00573-s001.zip › molecules-4099877-supplementary.pdf]

Novel Tyrosinase and α-Glucosidase Inhibitors: 1,3-Bisbenzylphenylphenol and Congeners as Whiten Agents Based on Natural Products

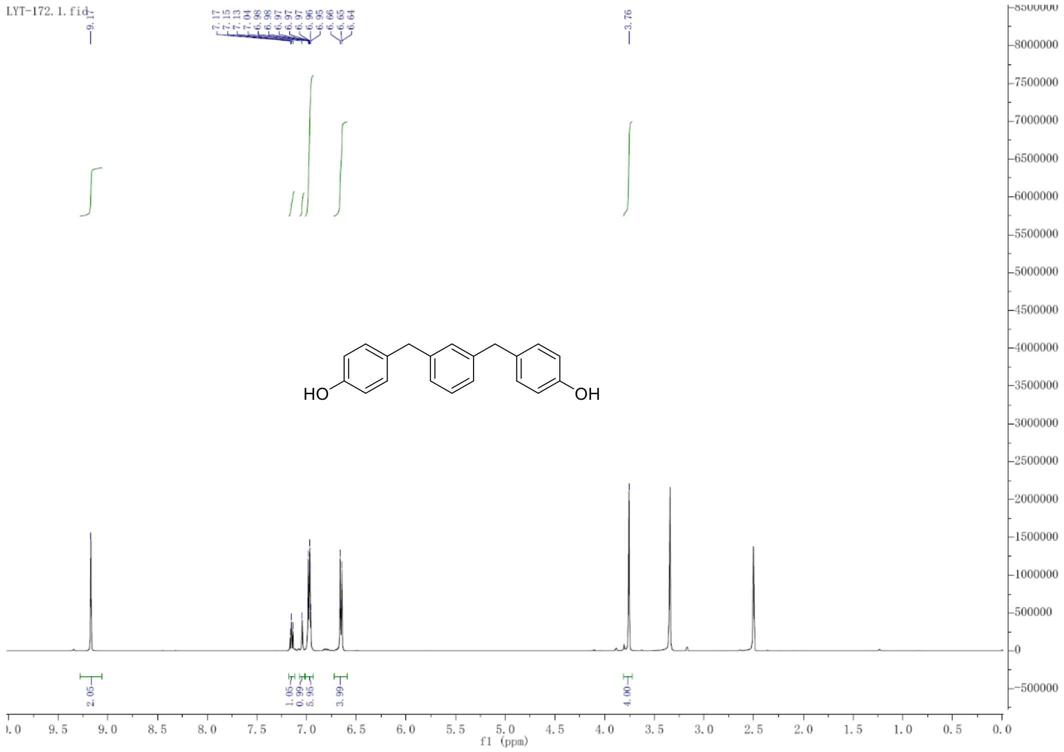

Figure S1. The ^{1}H NMR Spectra of compound 1 (600 MHz, CD_3OD).

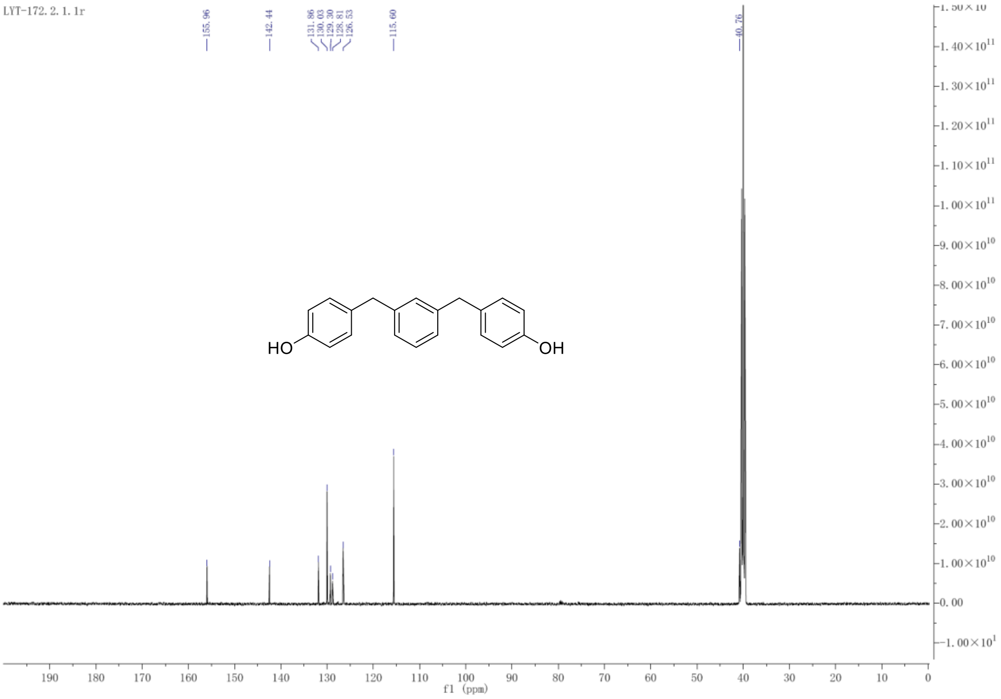

Figure S2. The ^{13}C NMR spectra of compound 1 (150 MHz, DMSO-d_6).

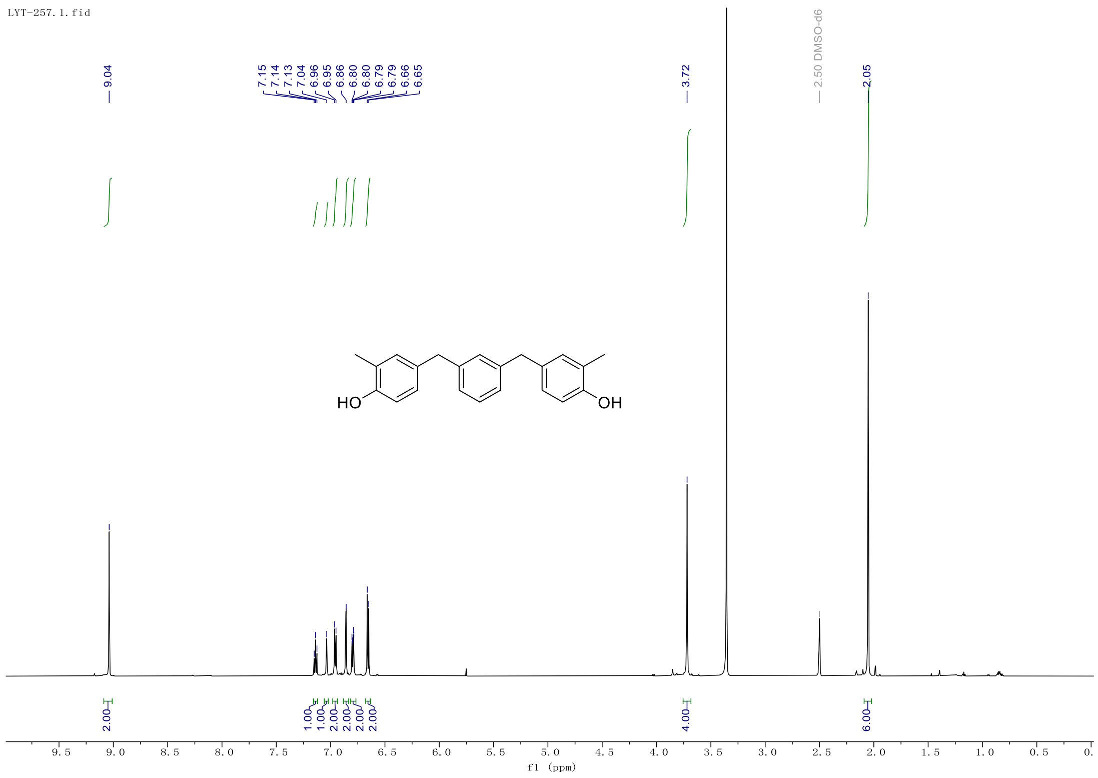

Figure S3. The ^{1}H NMR spectra of compound 2 (600 MHz, DMSO-d_6).

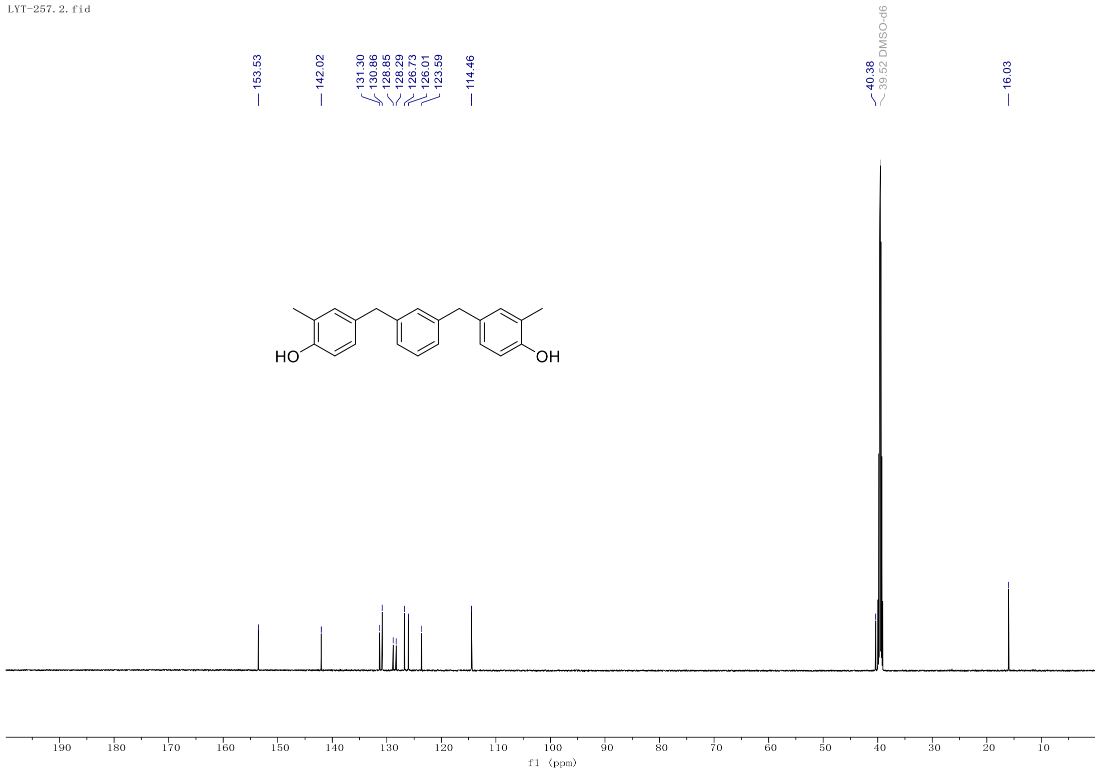

Figure S4. The ^{13}C NMR spectra of compound 2 (150 MHz, DMSO-d_6).

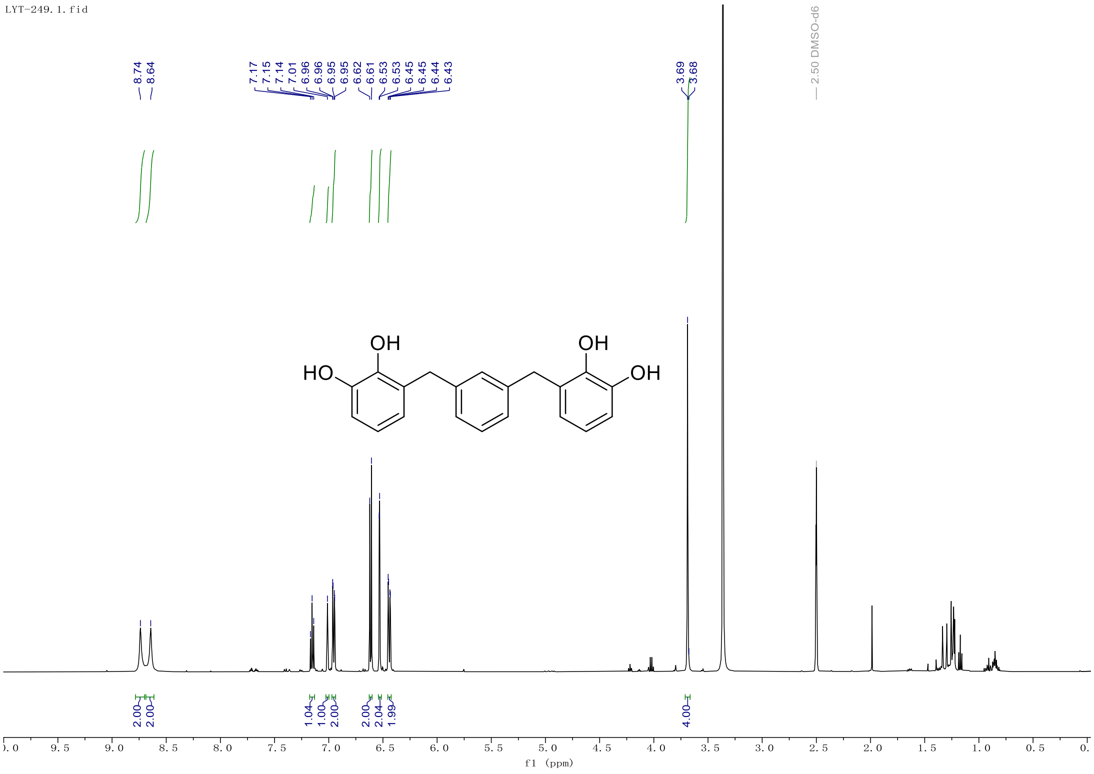

Figure S5. The ^{1}H NMR spectra of compound 3 (500 MHz, DMSO-d_6).

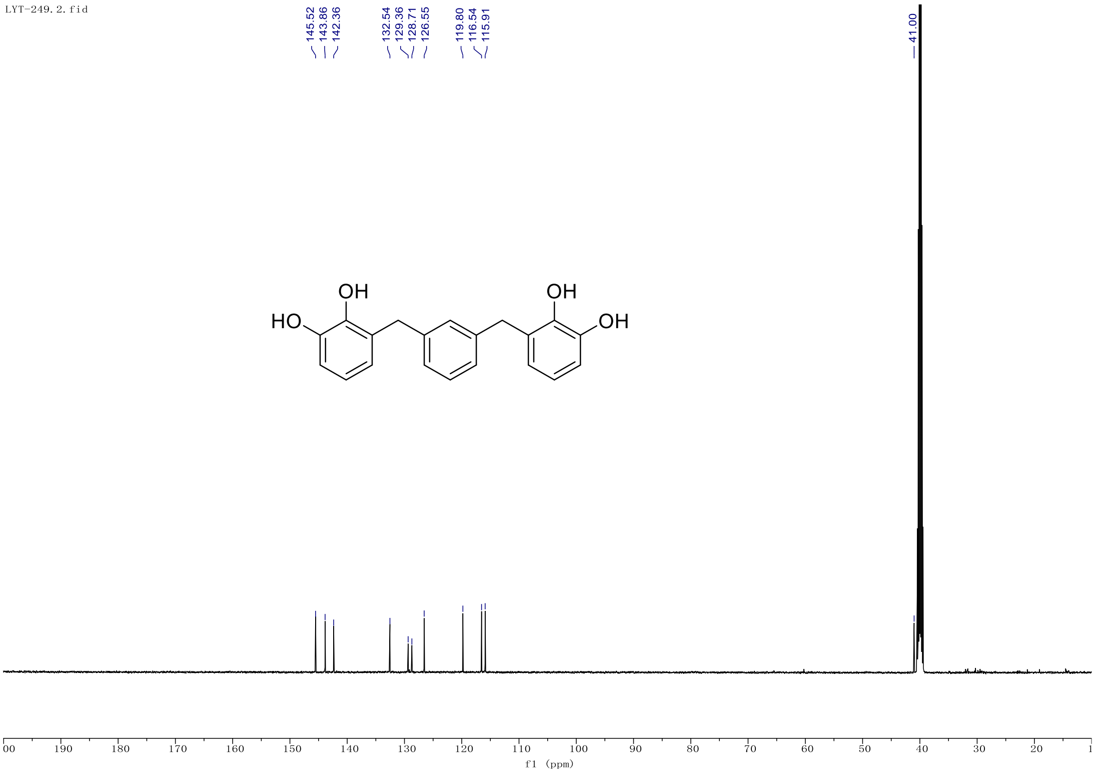

Figure S6. The ^{13}C NMR spectra of compound 3 (125 MHz, DMSO-d_6).
